# Astrocyte remodeling in the beneficial effects of long-term voluntary exercise in Alzheimer’s disease

**DOI:** 10.1186/s12974-020-01935-w

**Published:** 2020-09-15

**Authors:** Irina Belaya, Mariia Ivanova, Annika Sorvari, Marina Ilicic, Sanna Loppi, Hennariikka Koivisto, Alessandra Varricchio, Heikki Tikkanen, Frederick R. Walker, Mustafa Atalay, Tarja Malm, Alexandra Grubman, Heikki Tanila, Katja M. Kanninen

**Affiliations:** 1 A.I. Virtanen Institute for Molecular Sciences, University of Eastern Finland, FI-70211 Kuopio, Finland; 2 School of Biomedical Sciences and Pharmacy and the Priority Research Centre for Stroke and Brain Injury, The University of Newcastle, University Dr, Callaghan, NSW 2308 Australia; 3 Institute of Biomedicine, University of Eastern Finland, FI-70211 Kuopio, Finland; 4 Department of Anatomy and Developmental Biology, Monash University, Melbourne, Australia; 5 Development and Stem Cells Program, Monash Biomedicine Discovery Institute, Melbourne, Australia; 6 Australian Regenerative Medicine Institute, Monash University, Melbourne, Australia

**Keywords:** Alzheimer’s disease, Voluntary exercise, 5xFAD mouse, Behavior, BDNF, Astrocyte, GFAP, Morphology

## Abstract

**Background:**

Increased physical exercise improves cognitive function and reduces pathology associated with Alzheimer’s disease (AD). However, the mechanisms underlying the beneficial effects of exercise in AD on the level of specific brain cell types remain poorly investigated. The involvement of astrocytes in AD pathology is widely described, but their exact role in exercise-mediated neuroprotection warrant further investigation. Here, we investigated the effect of long-term voluntary physical exercise on the modulation of the astrocyte state.

**Methods:**

Male 5xFAD mice and their wild-type littermates had free access to a running wheel from 1.5 to 7 months of age. A battery of behavioral tests was used to assess the effects of voluntary exercise on cognition and learning. Neuronal loss, impairment in neurogenesis, beta-amyloid (Aβ) deposition, and inflammation were evaluated using a variety of histological and biochemical measurements. Sophisticated morphological analyses were performed to delineate the specific involvement of astrocytes in exercise-induced neuroprotection in the 5xFAD mice.

**Results:**

Long-term voluntary physical exercise reversed cognitive impairment in 7-month-old 5xFAD mice without affecting neurogenesis, neuronal loss, Aβ plaque deposition, or microglia activation. Exercise increased glial fibrillary acid protein (GFAP) immunoreactivity and the number of GFAP-positive astrocytes in 5xFAD hippocampi. GFAP-positive astrocytes in hippocampi of the exercised 5xFAD mice displayed increases in the numbers of primary branches and in the soma area. In general, astrocytes distant from Aβ plaques were smaller in size and possessed simplified processes in comparison to plaque-associated GFAP-positive astrocytes. Morphological alterations of GFAP-positive astrocytes occurred concomitantly with increased astrocytic brain-derived neurotrophic factor (BDNF) and restoration of postsynaptic protein PSD-95.

**Conclusions:**

Voluntary physical exercise modulates the reactive astrocyte state, which could be linked via astrocytic BDNF and PSD-95 to improved cognition in 5xFAD hippocampi. The molecular pathways involved in this modulation could potentially be targeted for benefit against AD.

## Background

Alzheimer’s disease (AD) is the most common neurodegenerative disorder pathologically characterized by accumulation of amyloid-beta (Aβ) plaques, formation of intracellular neurofibrillary tangles, neuronal loss, neuroinflammation, and oxidative stress [1]. Due to lack of broadly effective therapies, there is growing focus on modifiable lifestyle factors that may reduce disease risk or slow disease progression. Because it is known that a sedentary lifestyle is associated with impaired cognitive function and AD [2], one possible way to ameliorate AD pathology is regular physical exercise. Indeed, multifold benefits of regular physical exercise have been reported in both human and mouse models [3]. For instance, physical exercise has been shown to improve learning and memory by increasing long-term potentiation (LTP) and neurogenesis [4]. In addition, physical exercise is associated with structural and functional changes in the brain, promoting neuronal plasticity through increasing the levels of neurotropic and growth factors such as brain-derived neurotrophic factor (BDNF) [5]. Given that glial cells regulate many aspects of neuronal function and synaptic plasticity, the effects of physical exercise on glial cells may have broad implications for cognitive function, learning, and memory [6].

The exercise-induced cognitive improvements in AD mice is often associated with modulation of neuroinflammation [7]. Glial activation is a well-described feature of the AD brain [8], manifested as altered cell morphology [9, 10] and function [11]. Both astrocytes and microglia are implicated in AD progression [1] and recent cell type-specific gene expression data have undoubtedly provided evidence for the strong involvement of astrocytes in human AD [12, 13].

The most commonly reported exercise-induced effects in astrocytes include morphological changes, together with elevation or suppression in the levels of glial fibrillary acidic protein (GFAP) [14–19] . The reported difference in GFAP levels may be explained partly by astrocyte heterogeneity and partly by differences in the type of exercise intervention. Most studies have used voluntary wheel running, although daily access periods, duration of exercise, and age at the start of intervention have varied considerably among studies. Additionally, various murine models of AD have been used that display considerable variation in type and onset of pathology. The positive effect of physical exercise on the 5xFAD mouse model remains poorly investigated, although 5xFAD is a useful AD model with rapid Aβ plaque deposition starting from 2 months of age [20]. In particular, the impact of voluntary physical exercise on astrocyte-specific responses requires further clarification. Although astrocytes are important in AD progression and pathology, limited information exists on their role in exercise-mediated cognitive improvement.

We therefore tested the hypothesis that voluntary running exercise can improve cognitive deficits in the 5xFAD mouse model via astrocytic modulation. We assessed simultaneously a wide range of pathological features including neurogenesis, inflammation, AD pathology, synapses, and neurotrophic factors. The involvement of astrocytes in exercise-induced neuroprotection was assessed by a panel of extensive morphological, histological, and biochemical assays.

## Methods

### Experimental design

We used male transgenic 5xFAD (*n* = 41) mice carrying human amyloid precursor protein (APP) with the APP Swedish, Florida, and London mutations and human presenilin-1 (PSEN1) including the M146L and L286V mutations, driven by the mouse Thy1 promoter [21] and their wild-type (WT, *n* = 41) littermates on the JAXC57BL/6J background. Mice were housed in controlled temperature, humidity, and 12:12-h light-dark cycle, and had *ad libitum* access to food and water. This study was carried out in accordance with the Council of Europe Legislation and Regulation for Animal Protection and was approved by the National Animal Experiment Board of Finland.

At 6 weeks of age, mice were randomly divided into four groups: WT-Sedentary (WT-SED, *n* = 21), WT-Exercised (WT-EXE, *n* = 20), 5xFAD-Sedentary (5xFAD-SED, *n* = 21), and 5xFAD-Exercised (5xFAD-EXE, *n* = 20). The mice from WT-SED and 5xFAD-SED groups were housed in individual cages. The mice from WT-EXE and 5xFAD-EXE groups were housed in the same size individual cages, and had free access to running wheels (diameter 24 cm, Technoplast, Italy) for 6 months; the counters (Sigma, Germany) were attached to each cage to monitor the running distance and time for individual mice on weekly bases. In addition, body weight was measured for all mice weekly throughout the study. Two days before sacrificing, running wheels were removed from the cages of exercised mice to avoid any acute effects of running.

### Behavioral testing

Nest building tests were performed for all mice at the age of 7 months to assess the ability of animals to build a nest—a basic rodent activity related to hippocampal function [22]. A paper towel (22 × 22 cm, Katrin PLUS, Mediq) was placed inside the home cage of each mouse, and after 24 h, the built nest was photographed and removed from the cage. Built nests were scored with the following points: 0 point—untouched nest, 1—flat nest with few bites, 2—creased/moved and bitten nest, and 3—creased and bitten nest forming a crater inside the original nesting material. The person scoring the nest was blinded to the group of the mice.

To measure exploration activity vs. anxiety of mice, the open field test was performed as previously described [23]. At the age of 7 months, mice were individually placed in an empty white circular box (diameter 120 cm, height 22 cm), facing a wall, and allowed to freely explore the area inside the box for 10 min. Next, the tested mouse was placed back into the home cage; the box was cleaned with 70% EtOH and dried for the next mouse. Each mouse was recorded by video camera, and then the behavioral parameters, including duration in the center zone (80 cm diameter area, s), frequency of entries into the center zone, and latency to first entry in the center zone (s), were analyzed using EthoVision XT 7.1 video tracking software (Noldus Information Technologies, The Netherlands).

The elevated plus-maze test was performed at the age of 7 months as previously described [23] to assess exploration vs. anxiety level of mice. The mice were individually placed in the center of the plus-maze (two open arms without walls—30 × 5 cm, and two closed arms—30 × 5 × 20 cm, all painted black) facing closed arm, and allowed to freely explore the maze for 5 min. Subsequently, the tested mouse was placed back into the home cage; the plus-maze was cleaned with 70% EtOH and dried for the next mouse. Each mouse was recorded by video camera, and then behavioral parameters including duration in the open arms (s), and frequency of entries into the open arms, were analyzed using EthoVision XT 7.1 video tracking software (Noldus Information Technologies, The Netherlands). Only those mice that made more than five entries into different arms during the test were included in the analyses.

Spatial learning and memory were evaluated by the Morris water maze (MWM) test at the age of 7 months by using a testing protocol, described in detail previously [24]. Before the test phases, mice were pre-trained to find a submerged transparent platform 10 × 10 cm inside the white circular pool (diameter 120 cm, height 22 cm) filled with room temperature water, but within a limited swimming area (70 × 13 cm). Three days later, the MWM test began, composed of acquisition and probe phases. The acquisition phase consisted of five acquisition trials per day over 5 days from four different starting points, which were randomly chosen within the pool area. During the acquisition phase, the mouse was placed inside the pool, facing the wall, and each trial continued until the mouse reached the escape platform, or 60 s if the mouse was not able to find the platform. On the fifth day, a probe test was performed, during which the mouse was placed inside the swimming pool without the escape platform for 60 s to assess search bias as an indication of memory. During the test, mice rested for approximately 5 min in a warm cage with a cotton towel. The swimming path of each mouse was recorded by an overhead video camera, and parameters including speed (cm/s), escape latency (s), time in the wall zone (15 cm radius from wall area, s) for acquisition trials, as well as latency and distance to platform zone (30 cm radius area in previous platform location, s) for the probe trial were analyzed using EthoVision XT 7.1 video tracking software (Noldus Information Technologies, The Netherlands).

### Tissue collection

Mice were deeply anesthetized with tribromoethanol (Sigma-Aldrich, USA), and then transcardially perfused with heparinized saline before collection of brains. The left brain hemisphere was immersion fixed with 4% paraformaldehyde in 0.1 M phosphate buffer (pH 7.4) for 22 h followed by incubation in 30% sucrose solution for 48 h, prior to snap freezing in liquid nitrogen and storage in − 70 °C until cryosectioning. The hippocampus was isolated from the right brain hemisphere, snap frozen in liquid nitrogen, and stored at − 70 °C until further use. The same animals were used for the biochemical assays such as, Real-Time PCR, Western blot and cytokine bead array, and immunohistochemistry.

### Protein and RNA isolation

Frozen hippocampal samples (*n* = 5–7/group) were manually homogenized in eight volumes of lysis buffer pH 7.4 containing 20 mM Tris, 250 mM sucrose, 0.5 mM EDTA, 0.5 mM EGTA, 4% (v/v) protease, and 1% (v/v) phosphatase inhibitor cocktails (Sigma-Aldrich, USA). Cytosolic extracts from the tissues were centrifugated at 5000×*g* for 10 min at + 4 °C. The supernatants were divided into aliquots and stored at − 70 °C. Protein concentrations were determined by using Pierce 660 nm Protein Assay Kit (ThermoFisher Scientific, USA). For total RNA isolation, one of the hippocampal aliquots was mixed with TRI Reagent (Sigma-Aldrich, USA), and RNA was isolated according to the manufacturer’s protocol. The concentration and purity of RNA was determined by using NanoDrop 2000 (ThermoFisher Scientific, USA); RNA samples with 260/280 ratios higher than 1.8 were selected.

### Quantitative real-time PCR

Before reverse transcription, genomic DNA was removed from all RNA samples with DNase I, RNase-free kit (ThermoFisher Scientific, USA) following the manufacturer’s instructions. Then, 1 μg of mRNA was reverse-transcribed using High Capacity cDNA Reverse Transcription Kit (ThermoFisher Scientific, USA). Quantitative real-time PCR (RT-PCR) was performed to measure relative mRNA expression of astrocyte and microglia marker genes, as well as the brain-derived neurotrophic factor (*Bdnf*) gene, by using StepOnePlus Real-Time PCR System (ThermoFisher Scientific, USA). TaqMan gene expression assays (ThermoFisher Scientific, USA) used for RT-PCR are listed in Supplementary Table 1. Results were normalized to the glyceraldehyde 3-phosphate dehydrogenase (*Gapdh*) endogenous control.

### Western blot

To measure BDNF, synaptic, and astrocytic markers protein level in hippocampal samples, 20 μg of proteins were used for Western blot (WB). Proteins were separated by 12% SDS-PAGE and transferred to polyvinylidene difluoride (PVDF) membranes (GE Healthcare Life science) by using Trans-Blot Turbo Transfer System (BioRad, USA). The membranes were blocked in 5% fat-free milk in PBST (phosphate-buffered saline with 0.2% of Tween-20) and incubated overnight at + 4 °C with following primary antibodies: rabbit anti-BDNF (1:1000), rabbit anti-synaptophysin (1:200), rabbit anti-postsynaptic density protein 95 (PSD-95, 1:1000), rabbit anti-glial fibrillary acidic protein (GFAP; 1:1000), rabbit anti-s100 calcium binding protein β (S100β; 1:5000), rabbit anti-glutamine synthetase (GS, 1:5000), and rabbit anti-aldehyde dehydrogenase 1 family, member L1 (ALDH1L1, 1:1000). The membranes were washed and incubated with secondary goat anti-rabbit antibodies conjugated with HRP (1:3000) for 2 h at room temperature. Proteins were visualized with SuperSignalTM West Pico PLUS Chemiluminescent substrate kit (Thermo Fisher Scientific, USA), and detected using BioRad ChemiDocTM Imaging System and quantified by using ImageLab software (BioRad, USA). Mouse anti-β-actin antibody (1:5000) was used as an internal standard, following incubation with donkey anti-mouse Cy5-conjugated antibodies (1:1000). The list of all antibodies with company names and catalog numbers is presented in Supplementary Table 1.

### Cytokine bead array

For determination of cytokine levels in hippocampal protein samples, the cytokine bead array (CBA) mouse inflammation kit (BD Biosciences, USA) was used according to the manufacturer’s protocol. For CBA, 30 μg of cytosolic proteins from hippocampal samples were used to measure concentrations of the following cytokines: IL-6, IL-10, MCP-1, IL12p70, IFNγ, and TNFα. Samples were run using CytoFlex S flow cytometer (Beckman Coulter, USA), and acquired data were exported and analyzed by FCAP Array v3.0 software (Soft Flow Inc., USA).

### Immunohistochemistry

Aβ plaque deposition, neuronal survival, neurogenesis, and activation/proliferation of microglia and astrocytes were assessed by immunostaining. Brains (*n* = 7–9/group) were cut in serial 20 μm sagittal sections, each 400 μm apart, using a cryostat (Leica Microsystems, Germany), and stored in anti-freeze solution at − 20 °C before staining. The sections were blocked in 10% normal goat serum (NGS) in PBST and incubated overnight at room temperature with the following antibodies: mouse anti-Aβ (1:1000), rabbit anti-neuronal nuclei (NeuN; 1:200 dilution), rabbit anti-doublecortin (DCX; 1:200), rabbit anti-ionized calcium binding adaptor molecule 1 (Iba1;1:250), rabbit anti-GFAP (1:500), and rabbit anti-S100β (1:200). Sections were washed and incubated for 2 h with suitable fluorescent secondary antibodies, goat anti-rabbit AlexaFluor 488 (1:200) or goat anti-mouse AlexaFluor 568 (1:500). Antibodies with company names and catalog numbers are listed in Supplementary Table 1. Hippocampal areas from three sections 400 μm apart were imaged for each mouse. Images were taken with × 10 magnification on Zeiss Axio Imager M2 using a digital camera (Axiocam, Zeiss, Germany) and ZEN software. Each image was stitched to avoid overlap of adjacent tiles and exported in TIFF format. Immunoreactivity was quantified using the ImageJ software (National Institute of Health, USA). The percentage of positively-stained area in the hippocampus and subiculum were measured for each brain section, and the average from three sections per mouse was reported.

### Colocalization analysis for BDNF and GFAP

For colocalization analysis, double staining for BDNF and GFAP was performed for 5xFAD mouse brain sections. Then, 20 μm sagittal sections were incubated overnight in rabbit anti-BDNF (1:150) and chicken anti-GFAP (1:2500) antibodies. Sections were washed and incubated for 2 h with suitable fluorescent secondary antibodies, goat anti-rabbit AlexaFluor 488 (1:250), or goat anti-chicken AlexaFluor 568 (1:250). For correlation analysis, hippocampal areas were imaged with × 40 magnification on Zeiss Axio Observer inverted microscope with LSM800 confocal module (Zeiss, Germany) and ZEN software. Before the analysis, the red and green images were converted to 8-bit format and correlation of two proteins was measured by the JACoP plugin in ImageJ software (National Institute of Health, USA). For assessing the colocalization, the Pearson’s correlation coefficient (Pr; degree of correlation between two colors), overlap coefficient, and Mander’s colocalization coefficient (MOC; the proportion of green pixels in red channel) were used, which were generated by the JaCoP 2 plugin.

Furthermore, quantitative colocalization analysis was performed using GFAP and BDNF images taken from hippocampal areas with × 10 magnification on Zeiss Axio Imager M2 using a digital camera (Axiocam, Zeiss, Germany) and ZEN software. Each image was stitched to avoid overlap of adjacent tiles and exported in TIFF format. Areas covered by green (BDNF) pixels were quantified in red (GFAP-positive astrocytes) pixels by Apoptosis correlator plugin in ImageJ software (National Institute of Health, USA) for each brain section, and the average from three sections per mouse was reported. The threshold was chosen for each channel and total number of overlapped pixels in both channels were calculated and normalized by the total number of pixels. Similar analysis was performed before for colocalization [25, 26].

### Morphological analysis of astrocytes

Morphological analysis of GFAP-positive astrocytes was performed on images taken from the same brain sections, which were used for single GFAP staining. In addition to measuring astrocyte morphology depending on plaque proximity, double staining for GFAP and Aβ was performed for 5xFAD mice brain sections. For the analysis, the hippocampal area from a single 20 μm sagittal section was imaged for each mouse. Images were taken with × 40 magnification on Zeiss Axio Imager M2 using a digital camera (Axiocam, Zeiss, Germany) and ZEN software. Each image was stitched to avoid the overlap of the adjacent tiles and exported in TIFF format. The number of GFAP-positive cells and quantitative analysis of cell morphological parameters were performed using digital modeling based on the MicroTrac analysis platform [27]. Cells that failed to be reconstructed correctly by the program were manually removed from the analysis. Example of images and representative GFAP-positive cells from each group of mice are presented in Fig. 6a.

### Statistical analyses

All values are expressed as mean ± SEM. Two-way analysis of variance (ANOVA) was used to examine main genotype and exercise effect between WT and 5xFAD mice followed by unpaired *t* test comparison in case of a significant interaction between two factors (genotype*exercise) to examine exercise effect for each genotype separately. For nonparametric data, a Mann–Whitney test was used to compare the values between two groups. Changes in weekly running distance and body weight as well as MWM data were evaluated using mixed-model ANOVA for repeated measures with genotype and exercise as between-subject factors. Statistical analyses were performed with GraphPad Prism 8.4.2 software (GraphPad Software Inc, USA) and SPSS 25.0 software (IBM, USA); a difference was considered significant with *p* < 0.05. The statistical outliers were determined with Grubbs’ test and removed from the analysis.

## Results

Mice in the voluntary exercise groups had free access to a running wheel from the age of 1.5 months to 7 months. The weekly weighing of mice revealed that the body mass of 5xFAD mice was significantly lower (*F*(1, 28) = 8.149, *p* = 0.006) than the body mass of WT mice in accordance to previous studies [28, 29]. The difference further increased as the mice aged (genotype*age interaction: *F*(24, 1872) = 6.068, *p* < 0.0001; Supplementary Figure 1A). Undertaking voluntary exercise slightly reduced the body mass of both WT and 5xFAD mice (*F*(1, 28) = 3.832, *p* = 0.054; Supplementary Figure 1A). The efficacy of exercise was assessed by monitoring the running distance of each mouse by employing running counters that were documented on a weekly basis (Supplementary Figure 1B). The weekly running distance was similar in both genotypes of mice (28 ± 1 km/week for WT and 30 ± 1 km/week for 5xFAD mice, *F*(1, 31) = 1.152, *p* = 0.29)

### Voluntary physical exercise recovered the impairment of nest building behavior and anxiety observed in 5xFAD mice

**Fig. 1:**
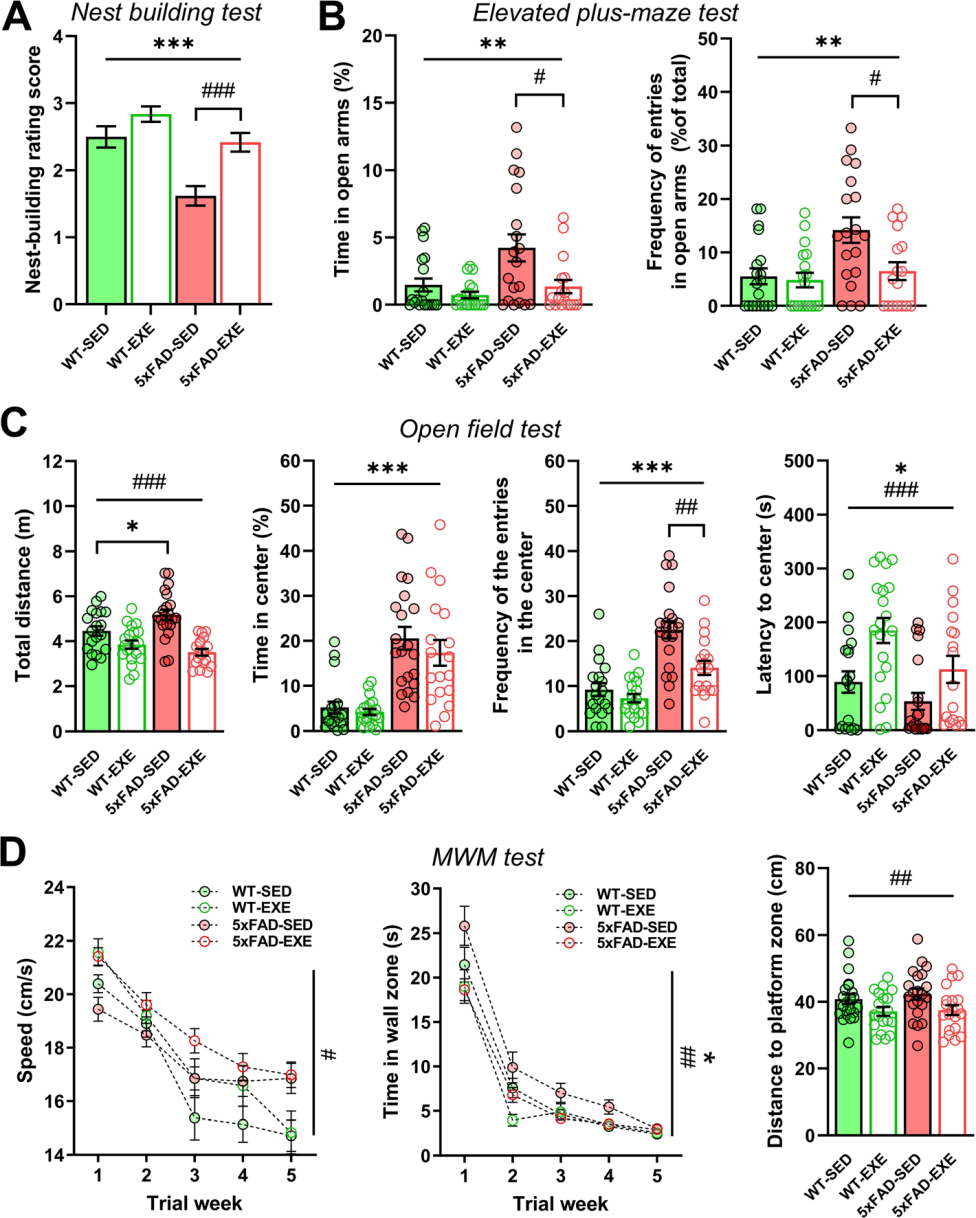
Voluntary physical exercise reverses cognitive impairment in 5xFAD mice. Nest building, elevated plus-maze, open field, and MWM tests were performed on WT-SED, WT-EXE, 5xFAD-SED, 5xFAD-EXE male mice at 7 months of age to assess effect of voluntary exercise on behavior. **a** Nest-building rating score. **b** Percentage of spent time and frequency of entries in open arms of elevated plus-maze test. **c** Total moved distance, time in the center, frequency of entries in the center, and latency to center entries in open field test. **d** Evolution of swimming speed and time spent in wall zone during 5-day acquisition trials and distance in platform zone during probe trial in MWM test. All data are presented as mean (±SEM). Two-way ANOVA to measure genotype and exercise effect between WT and 5xFAD mice followed by Mann-Whitney test (**a**,**b**) or unpaired *t* test (**c**) comparison in case of significant interaction between two factors (genotype*exercise); two-way ANOVA for repeated measures (**d**), with the test day as the within-subject factor. *n* = 20-22 per group. «*» Genotype effect, «#» exercise effect: *p < 0.05, **p < 0.01, ***p < 0.001, #*p* < 0.05, ##*p* < 0.01, ###*p* < 0.001

To evaluate the effect of voluntary physical exercise on typical behavioral features in mice, a nest building test was conducted at the age of 7 months. The sedentary 5xFAD (5xFAD-SED) mice demonstrated a significant impairment in the ability of build a nest, in comparison with sedentary WT (WT-SED) mice (Fig. 1a). The ANOVA revealed a significant main effect of genotype on nest score (*F*(1, 32) = 20.82, *p* < 0.0001). Voluntary physical exercise corrected this deficit (main effect of exercise: *F*(1, 32) = 16.08, *p* = 0.0001). The post-hoc test further revealed improvement in the exercised 5xFAD (5xFAD-EXE) mice (Mann–Whitney *U* (31) = 84, *p* = 0.0004) without having a significant effect on exercised WT (WT-EXE) mice (Mann–Whitney *U* (33) = 156, *p* = 0.08). These findings indicate that exercise is beneficial for nest building specifically for mice displaying AD-like pathology.

In the elevated plus-maze test, the 5xFAD-SED mice spent more time than WT-SED mice in the open arms. The ANOVA revealed a significant main effect of genotype on % time spent in open arms (*F*(1, 34) = 7.079, *p* = 0.0097) and on % number of visits to open arms (*F*(1, 34) = 8.281, *p* = 0.005; Fig. 1b). This is a characteristic anti-anxiety feature of this mouse line [21]. Voluntary physical exercise reversed this tendency in terms of % time spent in open arms (main effect of exercise: *F*(1, 34) = 8.115 *p* = 0.006) and % number of visits to open arms (main effect of exercise: *F*(1, 34) = 5.465, *p* = 0.02; Fig. 1). The post-hoc tests further demonstrated the significant reduction in the time spent in open arms in the 5xFAD-EXE mice (Mann–Whitney *U* (35) = 95.5, *p* = 0.03) without changes in WT-EXE mice (Mann–Whitney *U* (36) = 147, *p* = 0.46). The data demonstrate that exercise is beneficial for ameliorating anxiety level in 5xFAD mice.

### Voluntary physical exercise influenced locomotor activity and explorative behavior in both WT and 5xFAD mice

To investigate effect of voluntary physical exercise on locomotor activity and exploration in WT and 5xFAD mice, the open field test was performed at the age of 7 months. The ANOVA revealed no difference between the genotypes in the total distance traversed (*F*(1, 37) = 0.921, *p* = 0.34), whereas the main effect of exercise was significant (*F*(1, 37) = 32.94, *p* < 0.0001); there was significant genotype*exercise interaction (*F*(1, 37) = 7.199, *p* = 0.009). The post-hoc test further revealed that the 5xFAD-SED mice walked a longer distance than the WT-SED mice (*t*(31) = 2.302, *p* = 0.03), whereas exercised mice of both genotypes had significantly lower total distance values than sedentary mice (Fig. 1c). In accordance with the anti-anxiety phenotype [21], the 5xFAD mice spent more time in the center of the open field (main effect of genotype: *F*(1, 37) = 49.18, *p* < 0.0001), and entered the arena center significantly more often (*F*(1, 37) = 43.39, *p* < 0.0001) and with shorter latency (*F*(1, 37) = 6.618, *p* = 0.012) in comparison with WT mice (Fig. 1c). Interestingly, the number of entries to the arena center was significantly decreased (main effect of exercise: *F*(1, 37) = 11.60, *p* = 0.0011) and the latency to enter was significantly increased (*F*(1, 37) = 13.00, *p* = 0.0006) in both genotypes of mice after voluntary physical exercise (Fig. 1c). The post-hoc tests further demonstrated the significant reduction in the number of entries to the arena center in 5xFAD-EXE mice (*t*(31) = 3.336, *p* = 0.002) without changes in WT-EXE mice (*t*(31) = 1.132, *p* = 0.27). Together, these results indicate that exercise reduces exploratory activity in WT and 5xFAD mice.

### Voluntary physical exercise improved spatial memory deficits in 5xFAD mice

To investigate the effect of voluntary physical exercise on spatial learning and memory, the Morris water maze (MWM) test was performed at the age of 7 months. Spatial learning over 5 days of task acquisition was assessed by measuring a range of parameters that characterize the ability of a mouse to find a hidden platform. Because exercised mice swam significantly faster than sedentary mice (*F*(1, 28) = 5.101, *p* = 0.027; Fig. 1d), the time spent in the wall zone was chosen as a reliable parameter for learning assessment that is independent of the swimming speed. The 5xFAD-SED mice spent significantly more time (*F*(1, 38) = 4.253, *p* = 0.046) than WT-SED mice near the pool wall. Voluntary physical exercise reduced this wall clinging tendency (thigmotaxis) in 5xFAD-EXE mice (*F* = (1, 33) = 9.202, *p* = 0.004; Fig. 1d). During a probe trial, voluntary physical exercise significantly reduced the mean distance to platform zone in both WT and 5xFAD mice (main exercise effect: *F*(1, 28) = 8.106, *p* = 0.006; Fig. 1d). Moreover, exercise recovered latency to the platform zone in 5xFAD-EXE mice (Mann–Whitney *U* (33) = 123.5, *p* = 0.023), which was increased in 5xFAD-SED mice in comparison to WT-SED mice (Mann–Whitney *U* (39) = 118, *p* = 0.009; data not shown). These findings show that exercise induces improvements in hippocampal-dependent learning and memory.

### Voluntary physical exercise did not alter neurogenesis and neuronal survival in 5xFAD hippocampi

**Fig. 2:**
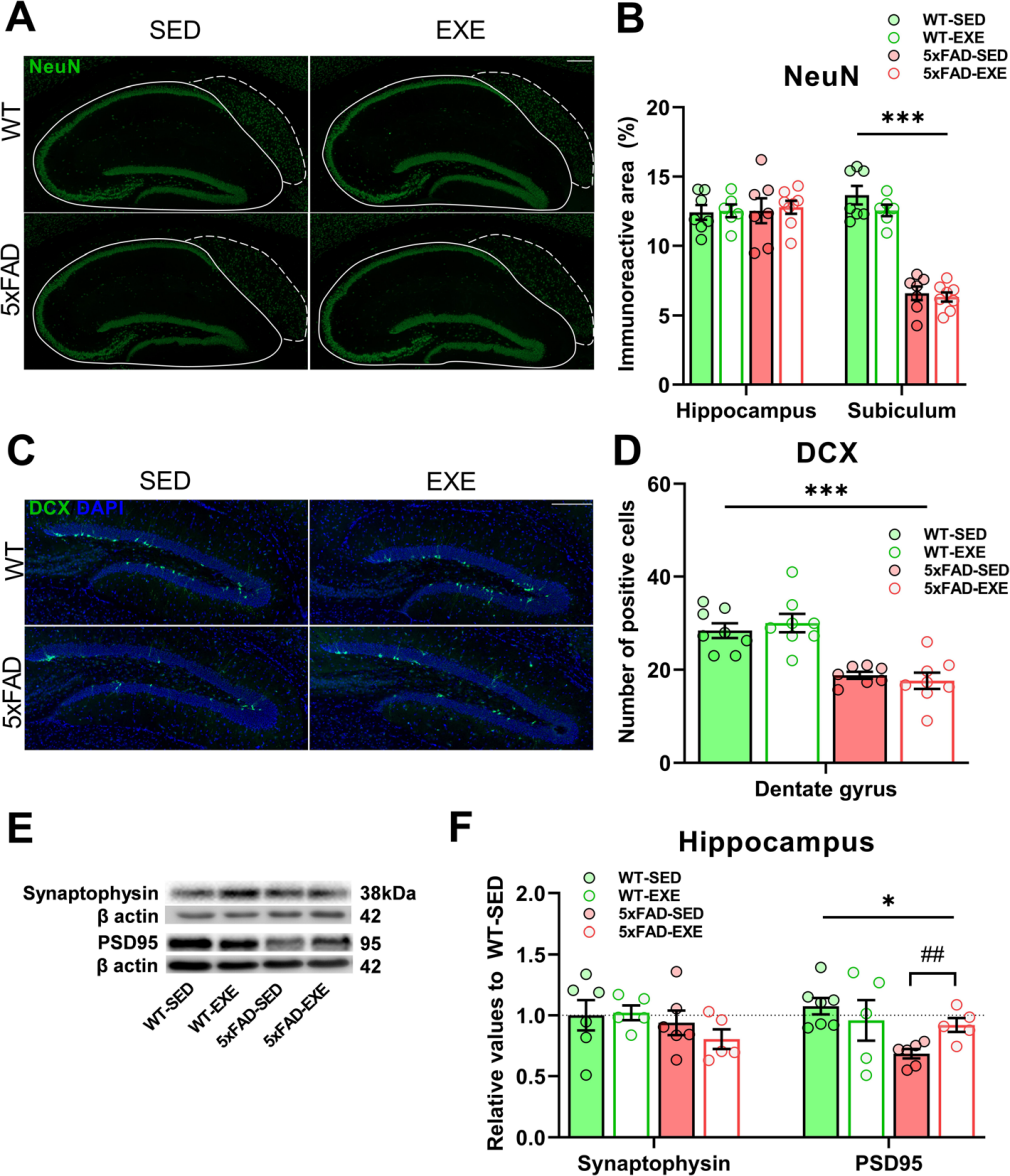
Voluntary physical exercise ameliorates AD-induced reduction of synaptic proteins without affecting neurogenesis and neuronal survival. **a** Representative image of NeuN-positive nuclei (green) in hippocampal areas of WT-SED, WT-EXE, 5xFAD-SED, 5xFAD-EXE mouse brains. Solid and dotted lines represent the quantified hippocampus and subiculum areas, respectively. Scale bars 200 μm. **b** Comparison of NeuN level in hippocampi and subiculum of WT and 5xFAD mice brain quantified by measuring the percentage of positive immunoreactive area. **c** Representative images of DCX-positive cells (green) and DAPI (blue) in hippocampal dentate gyrus of WT-SED, WT-EXE, 5xFAD-SED, 5xFAD-EXE mice. Scale bar 100 μm. **d** Comparison of DCX-positive cells number in dentate gyrus of WT and 5XFAD mice. **e** Representative WB of synaptic proteins synaptophysin, PSD-95 and β actin in hippocampal protein samples. **f** Analysis of the WB bands normalized to β actin and presented as relative values to WT-SED. All data are presented as mean (± SEM). Two-way ANOVA used to measure genotype and exercise effect between WT and 5xFAD mice followed by unpaired *t* test comparison in case of significant interaction between two factors (genotype*exercise). **a**–**d** *n* = 8/group, **e**, **f** *n* = 5–7/group. «*» Genotype effect, «#» exercise effect: *p < 0.05, ***p < 0.001, ##*p* < 0.01

Adult hippocampal neurogenesis was previously shown to be impaired in AD [30]. Thus to investigate neurogenesis and neuronal survival as potential mechanisms underlying the behavioral changes upon voluntary physical exercise, we use histochemistry. Immunostaining of brain sections for NeuN (Fig. 2a), a marker of mature neurons, revealed that NeuN was significantly reduced in the subiculum of 5xFAD mice compared to WT mice (main effect of genotype: *F*(1, 24) = 182.6, *p* < 0.0001), with no changes observed in the hippocampi (*F*(1, 24) = 0.09, *p* = 0.77; Fig. 2b). Voluntary physical exercise did not alter the NeuN level in WT or 5xFAD mice (subiculum: *F*(1, 24) = 1.932, *p* = 0.18; hippocampi: *F*(1, 24) = 0.08, *p* = 0.77). Hippocampal neurogenesis was assessed by immunostaining for the immature neuronal marker DCX in the dentate gyrus (DG) (Fig. 2c). The number of DCX-positive cells in the DG was significantly reduced in 5xFAD mice when compared to WT mice (main effect of genotype: *F*(1, 27) = 46.16, *p* < 0.0001); Fig. 2d). However, no difference was observed in the number of DCX-positive cells between sedentary and exercised mice (main effect of exercise: *F*(1, 27) = 0.02, *p* = 0.89), indicating that the observed behavioral improvement occurring upon exercise is not linked to increased neurogenesis or amelioration of neuronal loss.

### Voluntary physical exercise ameliorated the reduction of synaptic protein PSD-95 in 5xFAD hippocampi

To assess the effect of voluntary physical exercise on synaptic proteins known to be altered in AD [31], WB was used to measure the protein levels of the presynaptic marker synaptophysin and the postsynaptic marker PSD-95 in WT and 5xFAD hippocampi (Fig. 2e). While there was no genotype (*F*(1, 18) = 1.967, *p* = 0.18) or exercise (*F*(1, 18) = 0.326, *p* = 0.57) effect observed in the hippocampal level of synaptophysin, a significant reduction in the expression of PSD-95 was found in the hippocampi of 5xFAD mice in comparison with WT mice (main effect of genotype: *F*(1, 19) = 5.786, *p* = 0.03; Fig. 2f); there was genotype*exercise interaction (*F*(1, 19) = 3.927, *p* = 0.06). The post-hoc test further revealed that voluntary physical exercise ameliorated this reduction of PSD-95 in the 5xFAD mice hippocampi (*t*(9) = 3.544, *p* = 0.006) without changes in WT mice hippocampi (*t*(10) = 0.732, *p* = 0.48).

### Voluntary physical exercise increased GFAP levels without affecting Aβ burden in 5xFAD brains

**Fig. 3:**
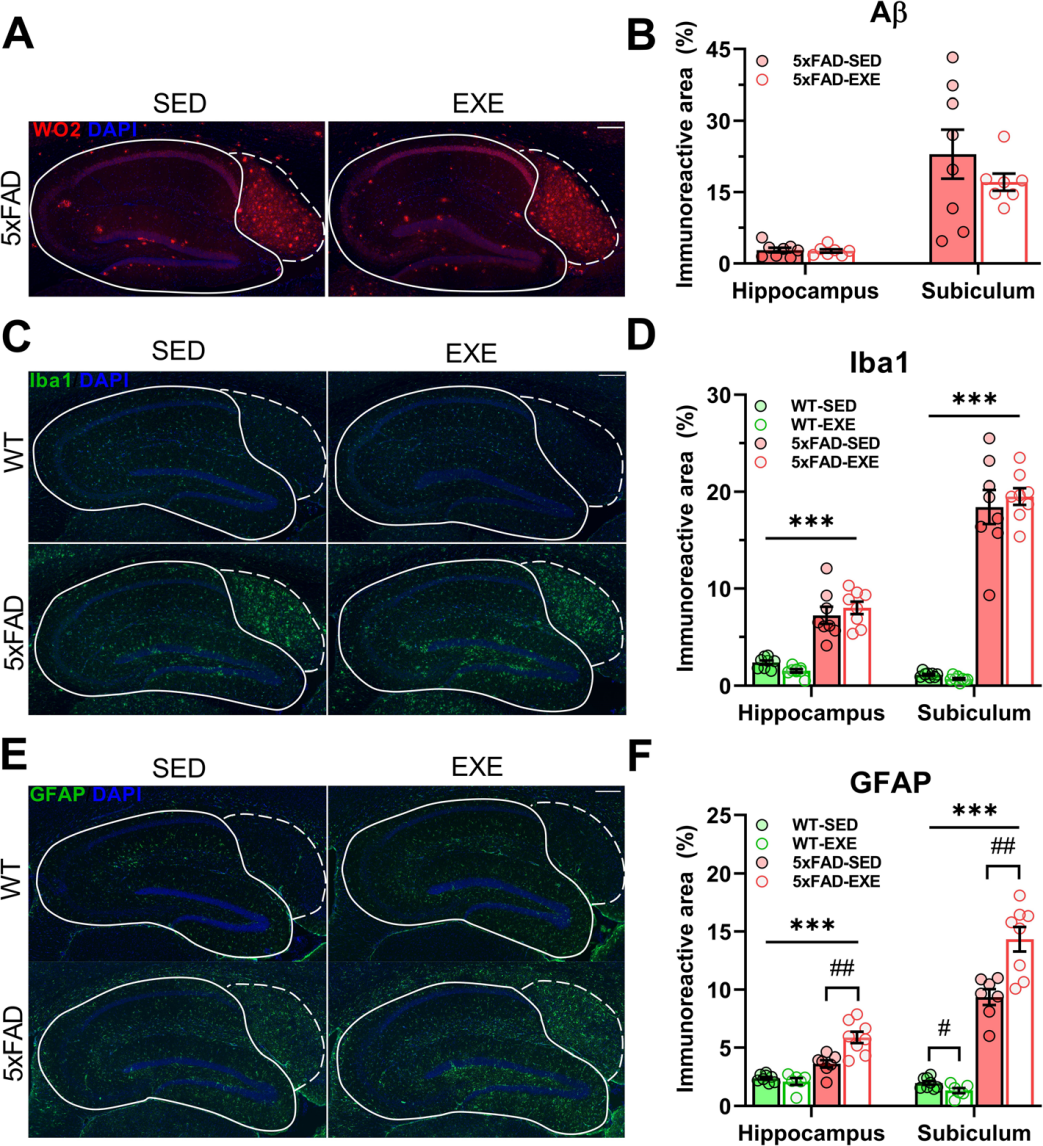
Effect of voluntary physical exercise on Aβ load and glial activation in hippocampi. Representative images of Aβ (**a**, red), GFAP-positive astrocytes (**c**, green), Iba1-positive microglia (**e**, green), and DAPI (blue) in the hippocampal area of WT-SED, WT-EXE, 5xFAD-SED, 5xFAD-EXE mice. Solid and dotted lines represent quantified hippocampi and subiculum areas, respectively. Scale bars: 200 μm. Aβ (**b**), Iba1 (**d**), and GFAP (**f**) levels were quantified by measuring the percentage of positive immunoreactive area in hippocampi and subiculum. All data are presented as mean (± SEM). Two-way ANOVA used to measure genotype and exercise effect between WT and 5xFAD mice followed by unpaired *t* test comparison in case of significant interaction between two factors (genotype*exercise). *n* = 8/group, «*» Genotype effect, «#» exercise effect: **p < 0.01, ***p < 0.001, #*p* < 0.05, ##*p* < 0.01

To investigate whether voluntary physical exercise affects Aβ plaque deposition and activation of glial cells, immunostaining was performed for Aβ, Iba1, a marker of activated microglia, GFAP, a marker of reactive astrocytes, and S100β, astrocytic marker. The level of Aβ, as determined by WO2 immunostaining, was not affected by voluntary physical exercise in the 5xFAD hippocampi (*t*(13) = 0.284, *p* = 0.78) or subiculum (*t*(13) = 1.016, *p* = 0.33; Fig. 3a, b). The expression of Iba1 was significantly increased in the hippocampi (main effect of genotype: *F*(1, 29) = 101.8, *p* < 0.0001) and subiculum (*F*(1, 29) = 336.7, *p* < 0.0001) of 5xFAD mice in comparison with WT mice, but not altered by voluntary physical exercise (hippocampi: *F*(1, 29) = 0.005, *p* = 0.95; subiculum: *F*(1, 29) = 0.110, *p* = 0.74; Fig. 3c, d). GFAP levels were significantly increased in the hippocampi (main effect of genotype: *F*(1, 25) = 52.23, *p* < 0.0001) and subiculum (*F*(1, 25) = 218.9, *p* < 0.0001) of 5xFAD mice in comparison with WT mice (Fig. 3e, f, Supplementary Table 3). Interestingly, the GFAP level was even further increased in both hippocampus and subiculum (*t*(13) = 3.679, *p* = 0.003 and *t*(13) = 3.847, *p* = 0.002, respectively) of exercised 5xFAD mice in comparison with 5xFAD-SED mice, whereas in the subiculum of WT mice GFAP was reduced after exercise (*t*(12) = 2.352, *p* = 0.04) in comparison with sedentary control. S100β levels were significantly increased in subiculum (main effect of genotype: *F*(1, 29) = 85.35 *p* < 0.0001) with no significant changes in hippocampi (*F*(1, 29) = 0.78, *p* = 0.38) of 5xFAD mice in comparison with WT mice (Supplementary Figure 2, Supplementary Table 3). Voluntary physical exercise did not alter the level of S100β in subiculum (*F*(1, 29) = 1.7 × 10^−7^, *p* = 0.99) and hippocampi (*F*(1, 29) = 0.10, *p* = 0.75) of mice from both genotypes. CBA measurements with hippocampal protein lysates (see Supplementary Table 2) demonstrated an increased level of the chemokine MCP-1 (19%, main effect of genotype: *F*(1, 20) = 11.41, *p* = 0.003) in 5xFAD mice in comparison with WT mice, and increased levels of the cytokine IL12p70 (54%, *t*(11) = 2.676, *p* = 0.02) in 5xFAD-SED mice in comparison with WT-SED mice. After voluntary physical exercise, the IL12p70 level was completely restored in hippocampi of 5xFAD-EXE mice (*t*(9) = 2.543, *p* = 0.03). These results indicate that voluntary physical exercise affects specifically the reactive astrocytes in 5xFAD mouse brains.

### Voluntary physical exercise selectively affected the GFAP-positive population of astrocytes

**Fig. 4:**
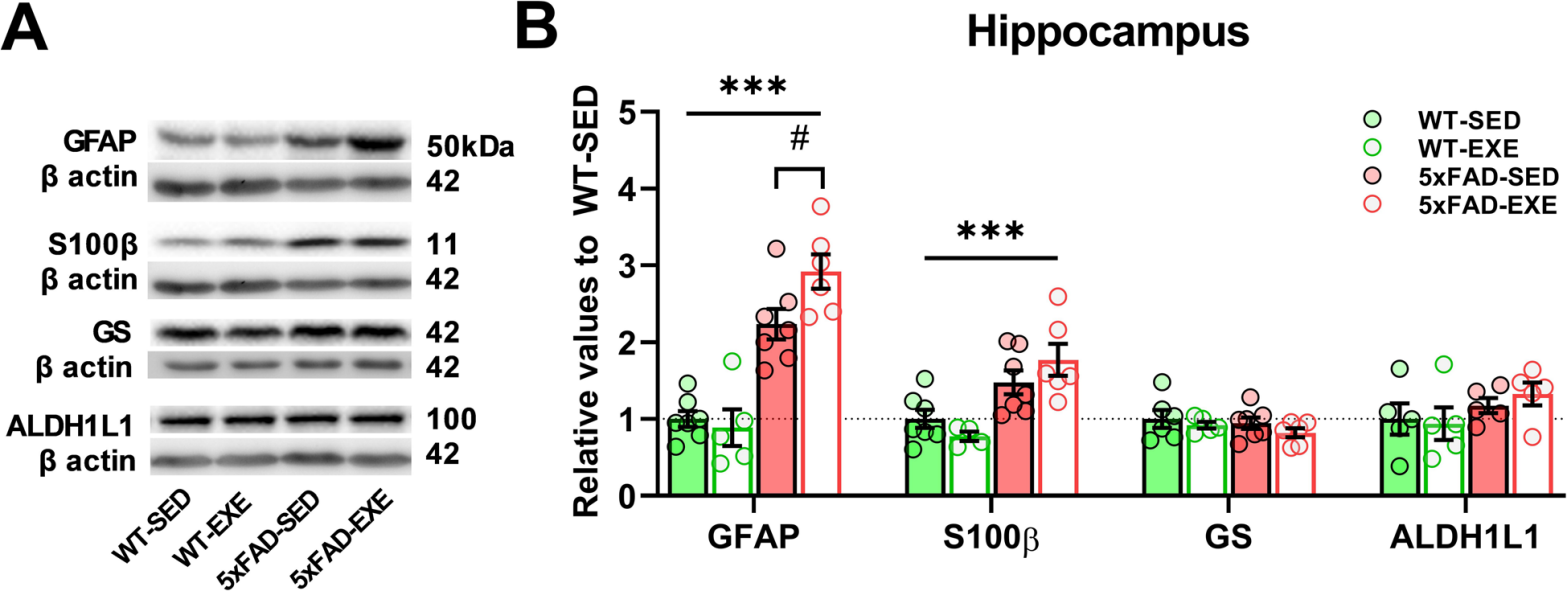
Effect of AD and voluntary physical exercise on level of astrocytic markers in hippocampi. **a** Representative WB of astrocytic markers GFAP, S100β, glutamine synthetase (GS), ALDH1L1, and β actin in hippocampal protein samples. **b** Analysis of the WB bands normalized to β actin. Data were normalized to WT-SED values and presented as mean (± SEM). Two-way ANOVA used to measure genotype and exercise effect between WT and 5xFAD mice followed by unpaired *t* test comparison in case of significant interaction between two factors (genotype*exercise). *n* = 5–7/group. «*» Genotype effect, «#» exercise effect: ***p < 0.001, #*p* < 0.05

Given the exercise-induced increase of GFAP immunoreactivity in 5xFAD brains, we next investigated whether other astrocytic markers such as S100β, glutamine synthetase (GS), and ALDH1L1 were affected by voluntary physical exercise in the hippocampus of 5xFAD mice. WB analyses revealed that the protein levels of GFAP (main effect of genotype: *F*(1, 21) = 72.42, *p* < 0.0001) and S100β (*F*(1, 20) = 21.03, *p* = 0.0002) were significantly upregulated in the hippocampi of 5xFAD mice compared to WT mice, whereas GS (*F*(1, 20) = 0.918, *p* = 0.35) and ALDH1L1 (*F*(1, 16) = 2.655, *p* = 0.12) protein levels remained unchanged (Fig. 4a, b). Interestingly, WB results demonstrated a similar increase in GFAP levels as shown by immunohistochemistry (Fig. 3e, f) when 5xFAD-EXE mice were compared to 5xFAD-SED mice (*t*(11) = 2.273, *p* = 0.04). In contrast, voluntary physical exercise did not significantly affect S100β, GS, or ALDH1L1 protein levels in WT or 5xFAD mice (Fig. 4b), which suggests that it is specifically the GFAP-positive astrocytes that respond to physical exercise in the 5xFAD mouse brain.

Recently, Liddelow et al. found two different types of reactive astrocytes: A1 astrocytes, which are harmful and related to neurodegenerative diseases including AD, and neuroprotective A2 astrocytes [32]. We decided to examine hippocampal mRNA expression level of A1/A2 astrocyte markers in 5xFAD and WT mice (Supplementary Table 3). RT-PCR analysis revealed increased mRNA expression of A1 markers, such as Fkbp5 (main effect of genotype: *F*(1, 19) = 6.088, *p* = 0.02), Srgn (*F*(1, 17) = 3.762, *p* = 0.07), as well as A2 marker S100A10 (*F*(1, 19) = 12.84, *p* = 0.002) in hippocampi of 5xFAD mice compared to WT mice. Voluntary physical exercise slightly reduced hippocampal mRNA expression level of A1 markers Serping1 (main effect of exercise: *F*(1, 18) = 3.306, *p* = 0.09) and Srgn (*F*(1, 17) = 1.826, *p* = 0.19) in exercised mice compared to sedentary, as well as A2 marker S100A10 (*t*(9) = 2.431, *p* = 0.04) in 5xFAD-EXE mice compared to 5xFAD-SED mice.

### Voluntary physical exercise ameliorated the reduction of BDNF in GFAP-positive astrocytes in hippocampi

**Fig. 5:**
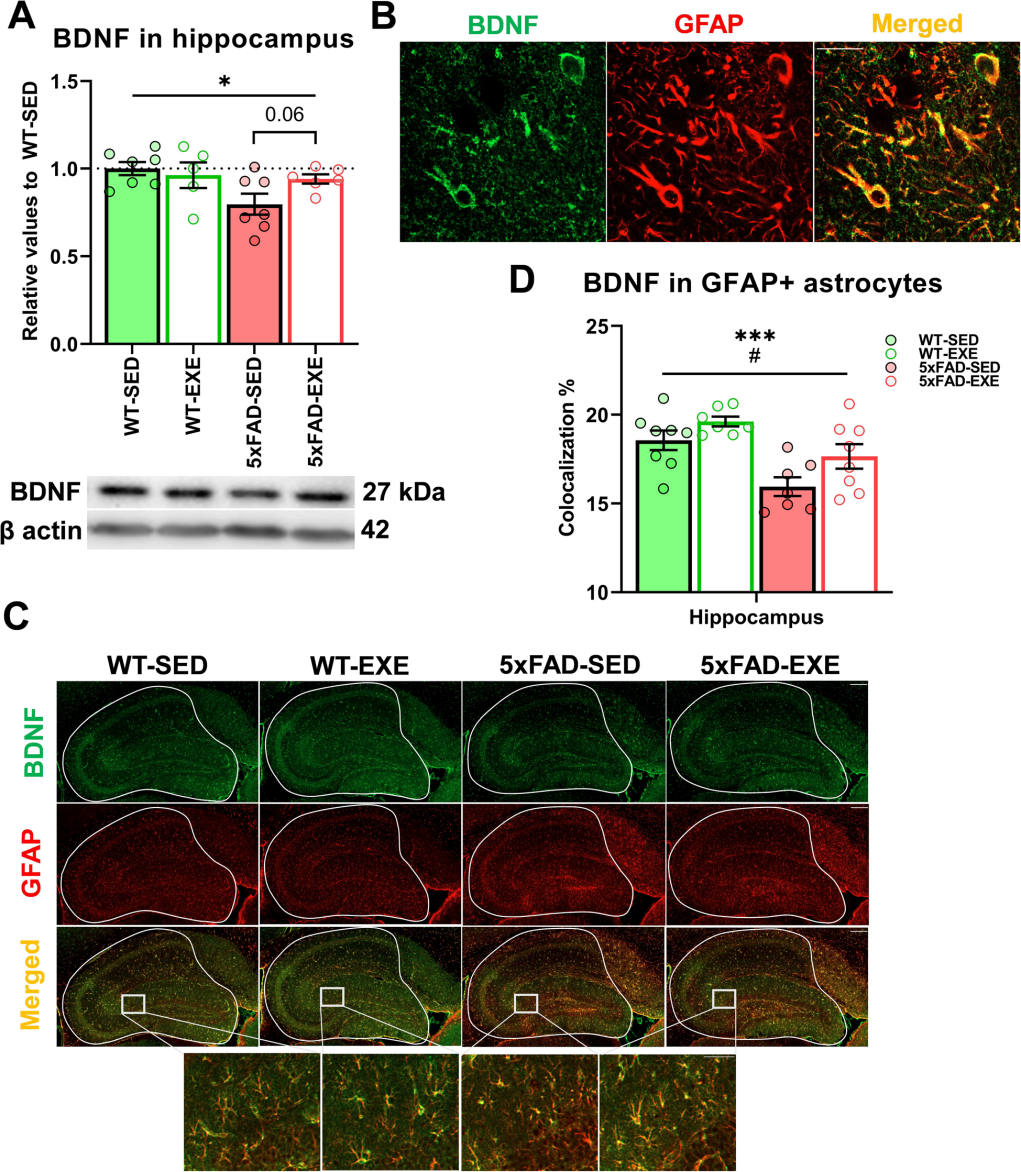
Effect of AD and voluntary physical exercise on BDNF level in hippocampi and GFAP-positive astrocytes. **a** Representative WB of BDNF and β actin in hippocampal protein samples and the analysis of the WB bands normalized to β actin. **b** Colocalization of BDNF (green) and GFAP (red) in hippocampus of WT mice. Scale bars: 20 μm. **c** Representative images of BDNF (green), GFAP-positive astrocytes (red), and Merged (yellow) in the hippocampal area of WT-SED, WT-EXE, 5xFAD-SED, 5xFAD-EXE mice. Solid lines represent quantified hippocampi area. Scale bars: 200 μm. The framed areas are magnified from merged images, scale bar 50 μm. **d** Level of colocalization (yellow) between BDNF (green) and GFAP (red) was quantified by measuring total number of overlapped pixels (yellow) in both channels normalized by the total number of pixels. All data are presented as mean (± SEM). Two-way ANOVA used to measure genotype and exercise effect between WT and 5xFAD mice followed by unpaired *t* test comparison in case of significant interaction between two factors (genotype*exercise). **a** *n* = 5–7/group, **d** *n* = 8/group. «*» Genotype effect, «#» exercise effect: *p < 0.05, ***p < 0.001, #*p* < 0.05

To assess the effect of voluntary physical exercise on BDNF, WB was used to measure the protein levels of BDNF in WT and 5xFAD hippocampi (Fig. 5a). We observed a reduction in the protein level of BDNF in the hippocampi of 5xFAD mice in comparison with WT mice (main effect of genotype: *F*(1, 21) = 4.88, *p* = 0.04), which was increased by 18% after voluntary physical exercise in 5xFAD-EXE mice (*t*(11) = 2.090, *p* = 0.06; Fig. 5a). In addition, double staining was performed in brain sections to assess the level of colocalization between BDNF and GFAP in WT and 5xFAD hippocampi (Fig. 5b). We performed correlation analysis between BDNF and GFAP and obtained the following correlation parameters: Pr = 0.49, overlap coefficient = 0. 93, and MOC = 0.51, which clearly indicate that hippocampal GFAP-positive astrocytes are expressing BDNF. Based on this finding, we decided to assess the level of BDNF in GFAP-positive astrocytes among all experimental groups in hippocampi (Fig. 5c). Colocalization analysis revealed that BDNF level was significantly lower in GFAP-positive astrocytes in 5xFAD mice in comparison with WT mice (main genotype effect: *F*(1, 26) = 17.35, *p* = 0.0003), whereas voluntary physical exercise corrected this deficit in hippocampi of both WT and 5xFAD mice (main effect of exercise: *F*(1, 26) = 6.335, *p* = 0.02; Fig. 5d). Altogether, these data demonstrate that voluntary physical exercise ameliorates the reduced BNDF level in hippocampi of 5xFAD mice, and specifically in GFAP-positive astrocytes.

### Voluntary physical exercise altered the morphology of GFAP-positive astrocytes in 5xFAD hippocampi

**Fig. 6:**
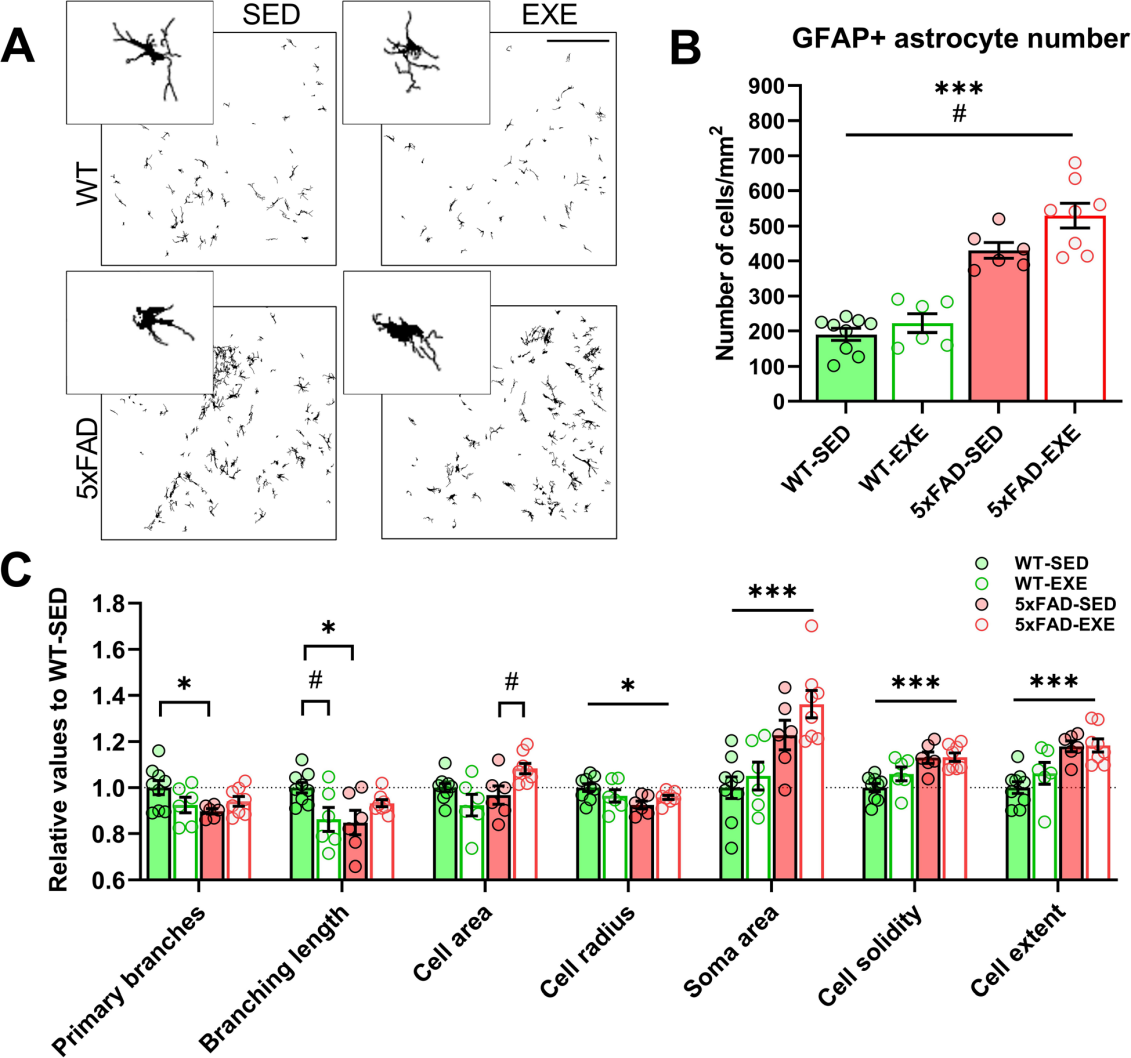
Effect of AD and voluntary physical exercise on morphology of GFAP-positive astrocytes in hippocampi. **a** Representative reconstructed images of GFAP-positive astrocytes in hippocampi of WT-SED, WT-EXE, 5xFAD-SED, 5xFAD-EXE mice using MicroTrac analysis software. Scale bar 100 μm. Small images display a representative single cell from each experimental group. **b** Number of GFAP-positive astrocytes per squared mm in hippocampi. **c** Analysis of GFAP-positive astrocyte morphological parameters such as number of primary branches, total branch length, cell area, cell radius, soma area, cell solidity, and extent. Parameters for each group were normalized to WT-SED values. All data are presented as mean (± SEM). Two-way ANOVA used to measure genotype and exercise effect between WT and 5xFAD mice followed by unpaired *t* test comparison in case of significant interaction between two factors (genotype*exercise). *n* = 8/group. «*» Genotype effect, «#» exercise effect: *p < 0.05, ***p < 0.001, #*p* < 0.05

To further examine the effects of voluntary physical exercise specifically on GFAP-positive astrocytes in the hippocampus, we employed a sophisticated morphological assessment using digital modeling based on the MicroTrac analysis platform as described [27]. Figure 6a depicts the typical morphology of GFAP-positive astrocytes in the brains of the different study groups. The analyses revealed a robust 2.4-fold increase in the number of GFAP-positive astrocytes in hippocampi of 5xFAD mice in comparison to WT mice (main effect of genotype: *F*(1, 25) = 104.1, *p* < 0.0001; Fig. 6b). Notably, voluntary physical exercise induced a further increase in the number of GFAP-positive astrocytes in 5xFAD and WT mice in comparison with sedentary mice (main effect of exercise: *F*(1, 25) = 6.004, *p* = 0.02). Interestingly, the number of S100β-positive astrocytes in hippocampi was constant for WT and 5xFAD mice (main genotype effect: *F*(1, 40) = 0.06, *p* = 0.81) and for sedentary and exercised mice (main exercise effect: *F*(1, 40) = 0.03, *p* = 0.87; Supplementary Figure 2).

Moreover, GFAP-positive astrocytes of 5xFAD mice displayed significant structural changes compared to WT mice (Fig. 6c), specifically smaller cell radius (4%, main effect of genotype: (*F*(1, 25) = 5.429, *p* = 0.03), enlarged soma area (28%, *F*(1, 25) = 21.8, *p* < 0.0001), increased cell solidity (13%, *F*(1, 25) = 21.3, *p* = 0.0001), and cell extent (18%, *F*(1, 25) = 21.94, *p* < 0.0001). The post-hoc tests further demonstrated that GFAP-positive astrocytes in 5xFAD-SED mice had significantly less primary branches (10%, *t*(13) = 2.629, *p* = 0.02) and shorter branch length (15%, *t*(13) = 2.980, *p* = 0.011) in comparison to WT-SED mice. Voluntary physical exercise partly restored these branching modifications in GFAP-positive astrocytes of 5xFAD mice, resulting in an increased number of primary branches (5%, *t*(12) = 1.648, *p* = 0.13) and increased branch length (14%, *t*(12) = 1.727, *p* = 0.11). Moreover, GFAP-positive astrocytes in 5xFAD-EXE mice had a significantly increased cell area (12%, *t*(12) = 2.687, *p* = 0.02) in comparison to 5xFAD-SED mice, whereas this parameter in 5xFAD-SED mice was unchanged (*t*(13) = 0.832, *p* = 0.42) when compared to WT-SED mice. Taken together, these results indicate that the presence of AD-like pathology induces robust alterations in GFAP-positive astrocytes, including an increase in the number of cells, concomitantly with soma hypertrophy and process atrophy. Voluntary physical exercise induced an increase in the cell area and increased the number of GFAP-positive astrocytes in the 5xFAD mouse brains.

**Fig. 7:**
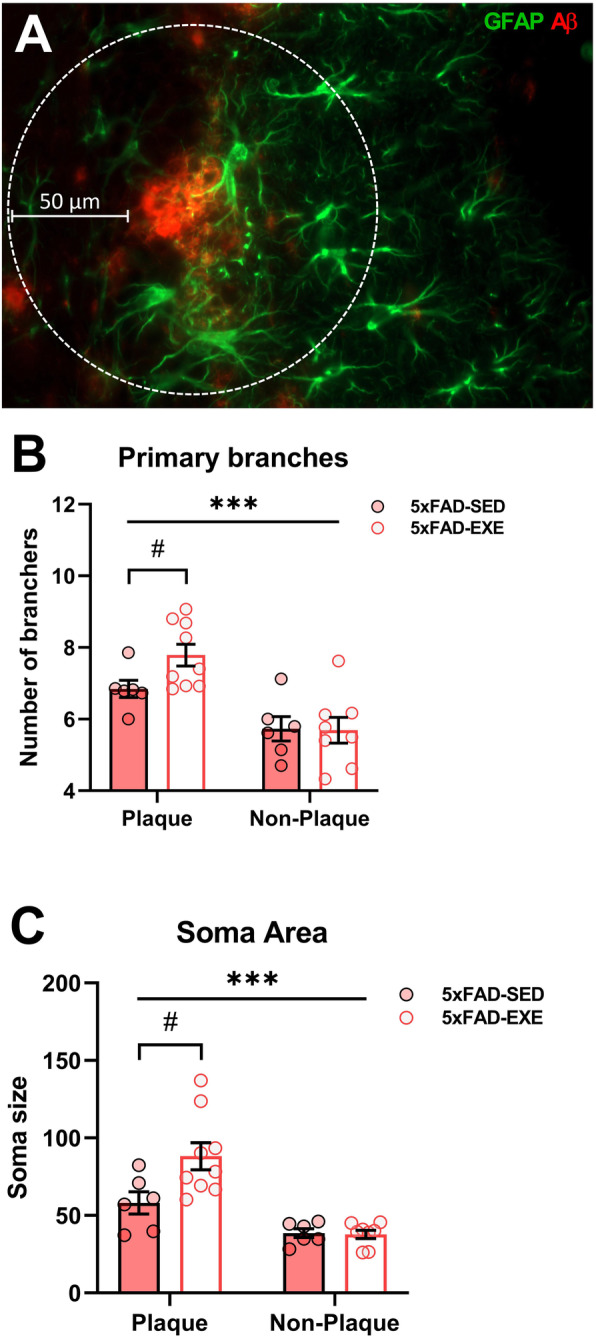
Voluntary physical exercise selectively affects plaque-associated GFAP-positive astrocytes in hippocampi. **a** Representative image of Aβ plaque (red) surrounded by a GFAP-positive astrocyte (green) in the hippocampus of a 5xFAD mice. Dotted circle displays a 50 μm area, within which GFAP-positive astrocytes were considered as plaque-associated. Number of primary branches (**b**) and soma area (**c**) were quantified for plaque-associated and distant GFAP-positive astrocytes in hippocampi of sedentary and exercised 5xFAD mice by using MicroTrac analysis platform. All data are presented as mean (± SEM). Two-way ANOVA used to measure exercise and plaque effect between 5xFAD-SED and 5xFAD-EXE mice followed by unpaired *t* test comparison in case of significant interaction between two factors (exercise*plaque). *n* = 8/group. «*» Plaque effect, «#» exercise effect: ***p < 0.001, #*p* < 0.05

To determine whether voluntary physical exercise affects all the GFAP-positive astrocytes, or specifically only the Aβ plaque-associated GFAP-positive astrocytes in 5xFAD mice, morphological analysis was performed on hippocampal sections double labeled for GFAP and Aβ. GFAP-positive astrocytes were considered as plaque-associated if they were located at a distance less than 50 μm from the edge of the plaque (Fig. 7a). The analyses demonstrated that the distance from the plaque had a dramatic effect on the morphology of GFAP-positive astrocytes: plaque-associated cells had significantly more primary branches (30%, *F*(1, 25) = 23.71, *p* < 0.0001), increased branch length (50%, *F*(1, 25) = 17.69, *p* < 0.0003), bigger cell radius (15%, *F*(1, 25) = 12.91, *p* = 0.0014), larger cell area (74%, *F*(1, 25) = 26.62, *p* < 0.0001), and enlarged soma (100%, *F*(1, 25) = 27.60, *p* < 0.0001; Supplementary Table 4). Voluntary physical exercise induced a significant increase in the number of primary branches (14%, *t*(13) = 2.240, *p* = 0.04) and soma size (52%, *t*(13) = 2.440, *p* = 0.03) of plaque-associated astrocytes in the hippocampi of 5xFAD mice, without affecting cells distant from the plaques (primary branches: *t*(13) = 0.650, *p* = 0.53; soma size: *t*(13) = 0.197, *p* = 0.85; Fig. 7b, c).

## Discussion

The aim of the current study was to expand the knowledge on the possible positive role of astrocytes in exercise-mediated benefits in AD. Utilization of the 5xFAD mouse model in a voluntary physical exercise intervention paradigm that begins well before Aβ plaque formation provides an opportunity to mimic and test the benefits of a healthy lifestyle on the attenuation and possible prevention of AD pathology. Voluntary physical exercise was chosen instead of treadmill exercise as the voluntary paradigm has been shown to be more tolerable and induce lower stress levels, demonstrated by measurements of serum corticosterone [33]. In agreement with existing studies [34, 35], we monitored the amount of exercise undertaken by the mice and discovered that the weekly running distance and time was 30 ± 1 km and 20 ± 1 h, respectively. The refinement of an appropriate exercise regime to effectively regulate the neuroinflammatory response in AD model mice may be of high relevance to the human population and should be considered in all studies using experimental animals.

In accordance with previous studies in 5xFAD mice [16, 20, 21, 28, 36], we demonstrated significant behavioral impairments occurring at the age of 7 months. The nest building test, used to assess species-typical behavior in rodents requiring planning and execution of a series of actions, is associated with impairment in neurotransmitter systems and hippocampus-dependent function [37]. In the current study, the 5xFAD-SED mice displayed a significant impairment in nest construction, which was corrected almost to the WT levels by voluntary physical exercise. Our finding is in agreement with the study of Walker et al., in which 4 months of voluntary exercise improved nest building in 6-month-old TgCRND8 mice [38]. Typical rodent behavior is characterized by avoiding open areas and high levels of anxiety, whereas 5xFAD mice show a robust anti-anxiety phenotype [21]. Our results demonstrated that voluntary physical exercise stabilized the anxiety level and attenuated the exploration behavior of 5xFAD mice. Previous studies have also shown a positive exercise effect on exploration and anxiety levels in different mouse models, including the 3xTG-AD mice [35]. One recent study, however, demonstrated opposing effects of voluntary exercise on exploratory behavior and anxiety in the 5xFAD mouse model [39]. Comparing our results to the study of Svensson et al. is difficult due to the absence of WT mice as a baseline in their results. The hyperactivity of AD mice has been established in previous studies [29], [40], and is demonstrated in this study by the greater distance traveled by sedentary 5xFAD mice in comparison to WT controls in the open field test. In line with a previous study [38], our results demonstrated that 6 months of wheel running significantly reduced exploratory activity of 5xFAD and WT mice in terms of total distance traversed. In addition to non-cognitive testing, the results of this study showed exercise-induced improvements in hippocampal-dependent learning and memory, as has been previously shown in various mouse AD studies [17, 35, 41, 42] and in patients with AD [43]. Based on previous literature, it is possible that voluntary physical exercise may stabilize AD-related behavioral impairments of structural changes in the hippocampus, regulation of the neurotransmitter system, or the expression of growth factors that regulate neurogenesis, synaptic plasticity, and inflammation [4, 44].

Given that cognitive dysfunction in AD is associated with neuronal and synaptic loss accompanied with impaired neurogenesis, we first assessed whether the positive effects of voluntary exercise on cognition could be linked with parameters related to neuronal function. Here, we demonstrated that at 7 months of age, the 5xFAD mice had a significant reduction in the number of DCX-positive cells in the hippocampal dentate gyrus, as has been reported to occur in several AD mouse models [45, 46]. In previous studies, physical exercise and environmental enrichment (EE) have been shown to improve adult hippocampal neurogenesis in different AD mouse models [17, 47, 48]. Although a recent study in 5xFAD mice reported that voluntary exercise induces adult hippocampal neurogenesis [5], we did not observe this in our experiment. In the study of Choi et al., the number of mice in the running group was quite large (sedentary *n* = 18, exercise *n* = 43), and approximately 40% of the running mice had the same amount of DCX-positive cells as the sedentary mice. Nevertheless, the difference between our study and the work of Choi et al. could be explained by the difference in the age of mice used, as in that study, the authors detected increased neurogenesis after exercise in 5xFAD mice at 6 months of age, when mice have almost four times more DCX-positive cells in the dentate gyrus in comparison with 7-month-old 5xFAD mice. Firstly, the exercise-induced increase in the number of newborn neurons could depend on the stage of brain pathology and be more beneficial for neurogenesis at a younger age. Secondly, the positive effect of exercise could be diminished by single housing of the mice, which in turn has been shown to negatively impact on the hippocampal neurogenesis in AD mice [49]. On the other hand, previous studies support our finding of unaltered neurogenesis through a demonstration that EE and voluntary exercise only increases the number of newborn neurons in wildtype, but not in 5xFAD [50], APP/PS1KI [51], APP-23 [41, 52], or presenilin-1 mutant mice [53]. In [54], 6 months of EE and physical exercise restored impaired neurogenesis in 3xTG-AD mice, although the proportion of immature proliferating cells (DCX-positive) were unchanged irrespective of genotype or housing conditions. Based on these results, we can hypothesize that exercise may have effects on later stages of neuronal development, because DCX stained only immature neurons. Further in-depth studies are required in order to confirm the effect of long-term voluntary physical exercise on neurogenesis in AD.

Considering that reduced neurogenesis is closely associated with increased Aβ deposition [46], it is also possible that the lack of neurogenesis we observed upon voluntary exercise is linked to Aβ burden, which was also unaffected by the exercise intervention. In previous studies, exercise has been shown to differentially affect Aβ plaque load, either inducing Aβ reduction [5, 15, 17], or having no effect [40–42, 51, 55]. Recent studies in 5xFAD mice are in line with our observations, showing no effect of long-term voluntary running [39] or EE [16] on hippocampal Aβ load at 8 months and 12 months of age, respectively. Because different studies have used different mouse models with dissimilar speeds and degrees of Aβ deposition and pathology progression, it is difficult to directly compare the results of these studies. For example, male 5xFAD mice display Aβ plaque deposition starting from the age of 2 months [20], with fast progression and reaching a plateau at 10 months of age [28, 46], whereas other AD mouse models have slower plaque load progression [56]. We thus hypothesize that exercise may slow down the speed of plaque formation at a young age, but at later ages, this exercise-mediated reduction is no longer able to cope with the increased amount of Aβ accumulation known to occur during disease progression.

Neuronal survival, as measured by NeuN immunostaining, was reduced in the subiculum, but not hippocampi of 5xFAD mice, in line with a previous study [20]. Voluntary exercise did not affect NeuN levels in the current study. Exercise-mediated effects in NeuN levels have not previously been reported in this mouse model, but previous studies in APP/PS1 mice reported that the hippocampal neuronal density is increased after long-term treadmill exercise [15] and short-term voluntary physical exercise [17], suggesting that either the type of exercise, the duration of the exercise, or the animal model used affects NeuN levels. On the other hand, other study is in agreement with our results showing that EE accompanied with voluntary exercise did not rescue neuronal loss in hippocampi of APP/PS1KI mice [51]. The synaptic loss and cognitive decline observed in AD is often associated with reductions of synaptic proteins and BDNF, one of the major neurotrophins regulating neuronal survival and synaptic plasticity [57]. Previous studies been shown that hippocampal PSD-95, which plays an important role in synapse stabilization and plasticity [58], is reduced in an age-dependent manner in 5xFAD mice [20], and in various other AD models [59, 60]. In the present study, we observed a significant reduction in both BDNF and synaptic protein PSD-95 in 5xFAD hippocampi. These reductions were both reversed after 6 months of voluntary exercise. This finding is in agreement with details in a previous report [5]. Taken together, the findings of our study suggest that voluntary exercise partially improves synaptic function by increasing PSD-95 and BDNF in the hippocampi of AD mice with no effect on adult neurogenesis or neuronal survival.

One of the pathological hallmarks of AD is neuroinflammation, which is accompanied by activation of glial cells [61]. Astrocytes can transit to a reactive state in response to CNS damage, and this astrocytic reaction is associated with increased GFAP expression, altered expression of many genes, and concomitant morphological and functional alterations [1, 62, 63]. Based on the “inflammation hypothesis,” neuronal damage in AD is linked with brain inflammation mediated by glial activation with the increased expression of pro-inflammatory mediators and induced neurotoxic cascades [61, 64]. However, reactive astrocytes also play a neuroprotective role in AD by forming a glial scar around Aβ plaques and isolating damaged areas from the rest of the tissue [65]. In addition, Pomilio et al. have shown the presence of APP-related peptides inside astrocytes, together with significant increases of autophagic processes in GFAP-positive plaque-associated hippocampal astrocytes in PDAPP-J20 mice [66]. Moreover, attenuating astrocyte activation has been shown to accelerate plaque pathology and increase dystrophic neurites in APP/PS1 mice [67], indicating the protective effects of astrocyte activation. Currently, it remains elusive whether astrogliosis is beneficial or harmful and most likely depends on a variety of factors, including the phase of pathology.

GFAP is an intermediate filament, which is highly expressed in reactive astrocytes. As in previous studies [16, 68], our results revealed increased GFAP in 5xFAD hippocampi compared to WT mice. Furthermore, we report that exercised 5xFAD mice display an even higher level of GFAP compared to sedentary animals. This alteration is in line with studies showing that treadmill exercise induced an increase in hippocampal GFAP levels in healthy [18] and in a sporadic rat AD model [69]. In contrast, some studies have demonstrated that exercise has differential effects on astrocyte reactivity. For example, exercise has been shown to suppress astrocyte reactivity and reduce the number of hippocampal GFAP+ astrocytes in APP/PS1 mice [15, 17]. Some studies, on the other hand, have not reported EE- or exercise-dependent alterations in GFAP protein in cortex, DG, or thalamus of 5xFAD mice, whereas the GFAP mRNA level was significantly upregulated [16]. In a recent review focusing on neuroprotection in AD, increased astrocytic GFAP was mentioned as one of the neuromodulatory effects of physical exercise [70].

In the current study, a range of astrocytic markers were investigated for potential exercise-induced alterations in 5xFAD mice, yet only GFAP expression was altered significantly, indicating that the so-called reactive astrocyte is the most affected cell subtype. As others have proposed [71], our data suggest that enhanced GFAP expression in the AD brain is linked with a phenotypic change of pre-existing resting cells, not proliferation of new cells. Human postmortem studies have demonstrated that in contrast to microglia [72], the total number of astrocytes remains unaltered in AD, but the number of GFAP-positive astrocytes is increased [73]. Our work corroborates this by showing that number of S100β-positive astrocytes was unchanged for all mice, whereas 5xFAD-SED mice have two times more GFAP-positive astrocytes in the hippocampi than the WT-SED mice, and this number is further increased by 23% after physical exercise. Based on our findings, we propose that the exercise-induced increase of GFAP in 5xFAD hippocampi may be partly explained by an increased number of GFAP+ astrocytes manifested as a phenotypic shift of the cells to a more reactive state. We thus suggest that exercise could enhance the protective reaction of astrocytes during AD pathology.

In AD, increase expression of GFAP is accompanied with changes in astrocyte morphology. Reactive astrocytes accumulate around Aβ plaques and are associated with hypertrophy of the cell body and thickening of processes [66, 74, 75]. At the same time, senescent-looking astrocytes localizing far from Aβ plaques (> 50 μm) display atrophied cell somas and simplified processes in hippocampus and entorhinal cortex [76, 77]. The results of the current paper demonstrate that in comparison to WT mice, the hippocampal GFAP-positive astrocytes of 5xFAD mice have an enlarged soma area, solid shape, and atrophic branches, and most of these cells are plaque-associated. Importantly, the difference in astrocyte morphology depends on cell localization: GFAP-positive astrocytes associated with plaques have distinct morphological alterations suggestive of reactive properties in comparison to distant cells.

Prior studies have also linked physical exercise and EE to altered astrocyte morphology [14, 18, 78, 79], yet little is known about exercise-induced morphological alterations of astrocytes in the AD brain. Here, we demonstrate that voluntary physical exercise significantly increases the soma area and number of primary branches in the 5xFAD hippocampi. Further analysis revealed that exercise targets specifically the astrocytes associated with Aβ plaques, whereas cells distant from the plaques remain unchanged. Our results are in line with previous study demonstrating that exercise and EE induces an increase in cell and soma surface of GFAP-positive astrocytes in the hippocampi of 12-month-old 3xTG mice [19]. Taken together, we propose that the exercise-induced increase of GFAP in 5xFAD hippocampi, in addition to the phenotypic change to a more reactive state, is a consequence of the morphological changes induced by exercise in plaque-associated astrocytes.

One mechanism that may explain our observed exercise-mediated alterations in GFAP-positive astrocytes and link these to improved behavioral outcomes is related to BDNF, which was significantly upregulated in GFAP-positive astrocytes in the hippocampi of 5xFAD-EXE mice. Astrocytes play a crucial role in synaptic plasticity by regulating synaptic transmission and synaptic structure [65] and BNDF and tyrosine receptor kinase B (TrkB) participate in synaptic function [80]. Alterations in the BDNF-TrkB system thereby affect key parameters such as synaptic plasticity, synaptic proteins including PSD-95, and memory function, which are all impaired in AD [81]. Evidence for the involvement of astrocytes in BDNF-mediated cognitive outcomes is numerous. For example, astrocytes expressing a truncated form of TrkB T1 respond to application of BNDF by releasing Ca^2+^ from intracellular space [82], astrocytes respond to increased BDNF by expression of TrkB receptors [83], astrocytes themselves produce the BDNF protein as it was shown in our study and in previous works [84, 85], and BDNF released from astrocytes is crucial for dendrite spine density and morphology [86]. Importantly, BDNF has been shown to regulate astrocytic morphology through TrkB-T1 receptors located specifically in GFAP+ astrocytes [26, 87]. Collectively, our data and the previously published reports suggest that the observed exercise-mediated increase in the number of GFAP-positive astrocytes together with their morphological changes in the 5xFAD hippocampi result from restoration of reduced BDNF level in GFAP-positive astrocytes and PSD-95 level in hippocampi in AD. These alterations, stimulated by voluntary physical exercise, are likely to stabilize LTP and thereby reduce the cognitive decline observed in the 5xFAD mice.

## Conclusions

This paper highlights the importance of non-neuronal cells in the underlying benefits of voluntary exercise, providing additional insight into the significance of astrocytes as responders to physical exercise. Long-term voluntary physical exercise modulated the number of GFAP-positive astrocytes and the morphology of Aβ plaque-associated astrocytes in the hippocampi of 5xFAD mice. The molecular pathways involved in this modulation could potentially be targeted as a therapeutic strategy against AD.

## Supplementary information

**Additional file 1: Supplementary Figure 1**. Body mass and running activity evolution of WT and 5xFAD mice as measured on a weekly basis. (A) 5xFAD mice have significantly lower body weight than WT mice, whereas exercise slightly reduces body weight in all mice (**p <0.01, ^#^*p* = 0.054). (B) Mice had free access to a running wheel from 1.5 to 7 months of age. The running distance was monitored for each mouse by using running counters. No significant differences were observed in the running distance between WT and 5xFAD mice (p = 0.166). Data is presented as mean ± SEM, one-way and two-way ANOVA for repeated measures, *n* = 20-22/group. « * » genotype effect, **p<0.01, « # » exercise effect. **Supplementary Figure 2**. Effect of voluntary physical exercise on S100β-positive astrocytes in hippocampi. Representative images of S100β-positive astrocytes (A, green) and DAPI (blue) in the hippocampal area of WT-SED, WT-EXE, 5xFAD-SED, 5xFAD-EXE mice. Solid and dotted lines represent quantified hippocampi and subiculum areas, respectively. Scale bars: 200 μm. S100β (B) levels were quantified by measuring the percentage of positive immunoreactive area in hippocampi and subiculum. Number of S100β -positive astrocytes per squared mm in hippocampi (C). All data are presented as mean (±SEM). 2-way ANOVA used to measure genotype and exercise effect between WT and 5xFAD mice. n=8/group, « * » genotype effect: ***p<0.001. **Supplementary Table 1**. List of antibodies and TaqMan primers used in this study. **Supplementary Table 2**. Effect of AD and voluntary physical exercise on cytokine levels in the hippocampus. Data are presented as mean (±SEM). 2-way ANOVA was used to measure genotype and exercise effect between WT and 5xFAD mice followed by unpaired *t* test comparison in case of significant interaction between two factors (genotype*exercise). « * » genotype effect, « # » exercise effect, « § » interaction: *p<0.05, **p<0.01, ^#^*p*<0.05, ^§^*p*<0.05. **Supplementary Table 3**. Effect of AD and voluntary physical exercise on mRNA expression of glial markers and BDNF in the hippocampus. Data are normalized by GAPDH C_T_ values and presented as mean (±SEM). 2-way ANOVA was used to measure genotype and exercise effect between WT and 5xFAD mice followed by unpaired *t* test comparison in case of significant interaction between two factors (genotype*exercise). « * » genotype effect, « # » exercise effect, « § » interaction: *p<0.05, **p<0.01, ***p<0.001, ^#^*p*<0.05, ^§^*p*<0.05. **Supplementary Table 4**. Morphological analysis of plaque-associated and non-plaque-associated GFAP-positive astrocytes in hippocampus of 5xFAD mice. Data are presented as mean (±SEM). 2-way ANOVA was used to measure plaque-association and exercise effect between sedentary (SED) and exercised (EXE) mice followed by unpaired *t* test comparison in case of significant interaction between two factors (plaque*exercise). « * » plaque effect, « # » exercise effect, « § » interaction: **p<0.01, ***p<0.01, ^#^*p*<0.05, ^§^*p*<0.05.

## Data Availability

The datasets used and/or analyzed during the current study are available from the corresponding author on reasonable request.

## Abbreviations

AD: Alzheimer’s disease
Aβ: Amyloid beta
ALDH1L1: Aldehyde dehydrogenase 1 family, member L1
ANOVA: Analysis of variance
APP: Amyloid precursor protein
BDNF: Brain-derived neurotrophic factors
CBA: Cytokine bead array
DCX: Doublecortin
DG: Dentate gyrus
EE: Environmental enrichment
EXE: Exercised
GFAP: Glial fibrillary acidic protein
GS: Glutamine synthetase
Iba1: Ionized calcium binding adaptor milecule 1
LTP: Long-term potentiation
MOC: Mander's colocalization coefficient
MWM: Morris water maze
NeuN: Neuronal nuclear antigen
Pr: Pearson's correlation coefficient
PSD-95: Postsynaptic density 95
RT-PCR: Real-time PCR
S100β: s100 calcium binding protein β
SED: Sedentary
TrkB: Tyrosine receptor kinase B
WB: Western blot
WT: Wild type
