# Supplementary material for: Astrocyte remodeling in the beneficial effects of long-term voluntary exercise in Alzheimer’s disease
Source: J Neuroinflammation. 2020 Sep 15;17:271. doi: 10.1186/s12974-020-01935-w (PMC7493971; doi:10.1186/s12974-020-01935-w)
Supplement: Supplementary file 1 — Additional file 1: Supplementary Figure 1. Body mass and running activity evolution of WT and 5xFAD mice as measured on a weekly basis. (A) 5xFAD mice have significantly lower body weight than WT mice, whereas exercise slightly reduces body weight in all mice (**p <0.01, #p = 0.054). (B) Mice had free access to a running wheel from 1.5 to 7 months of age. The running distance was monitored for each mouse by using running counters. No significant differences were observed in the running distance between WT and 5xFAD mice (p = 0.166). Data is presented as mean ± SEM, one-way and two-way ANOVA for repeated measures, n = 20-22/group. « * » genotype effect, **p<0.01, « # » exercise effect. Supplementary Figure 2. Effect of voluntary physical exercise on S100β-positive astrocytes in hippocampi. Representative images of S100β-positive astrocytes (A, green) and DAPI (blue) in the hippocampal area of WT-SED, WT-EXE, 5xFAD-SED, 5xFAD-EXE mice. Solid and dotted lines represent quantified hippocampi and subiculum areas, respectively. Scale bars: 200 μm. S100β (B) levels were quantified by measuring the percentage of positive immunoreactive area in hippocampi and subiculum. Number of S100β -positive astrocytes per squared mm in hippocampi (C). All data are presented as mean (±SEM). 2-way ANOVA used to measure genotype and exercise effect between WT and 5xFAD mice. n=8/group, « * » genotype effect: ***p<0.001. Supplementary Table 1. List of antibodies and TaqMan primers used in this study. Supplementary Table 2. Effect of AD and voluntary physical exercise on cytokine levels in the hippocampus. Data are presented as mean (±SEM). 2-way ANOVA was used to measure genotype and exercise effect between WT and 5xFAD mice followed by unpaired t test comparison in case of significant interaction between two factors (genotype*exercise). « * » genotype effect, « # » exercise effect, « § » interaction: *p<0.05, **p<0.01, #p<0.05, §p<0.05. Supplementary Table 3. Effect of AD and voluntary phy [file 12974_2020_1935_MOESM1_ESM.docx]

Supporting information for:

**Astrocyte remodeling in the beneficial effects of long-term voluntary exercise in Alzheimer’s disease**

Irina Belaya^1^, Mariia Ivanova^1^, Annika Sorvari^1^, Marina Ilicic^2^, Sanna Loppi^1^, Hennariikka Koivisto^1^, Alessandra Varricchio^1^, Heikki Tikkanen^3^, Frederick R. Walker^2^, Mustafa Atalay^3^, Tarja Malm^1^, Alexandra Grubman^4,5,6^, Heikki Tanila^1^, Katja M. Kanninen^1^

^1^ A.I. Virtanen Institute for Molecular Sciences, University of Eastern Finland, 70211, Kuopio, Finland,^2^ School of Biomedical Sciences and Pharmacy and the Priority Research Centre for Stroke and Brain Injury, The University of Newcastle, University Dr, Callaghan, NSW 2308, Australia, ^3^ Institute of Biomedicine, University of Eastern Finland, 70211 Kuopio, Finland, ^4^ Department of Anatomy and Developmental Biology, Monash University, Australia, ^5^ Development and Stem Cells Program, Monash Biomedicine Discovery Institute, Australia, ^6^ Australian Regenerative Medicine Institute, Monash University, Australia.


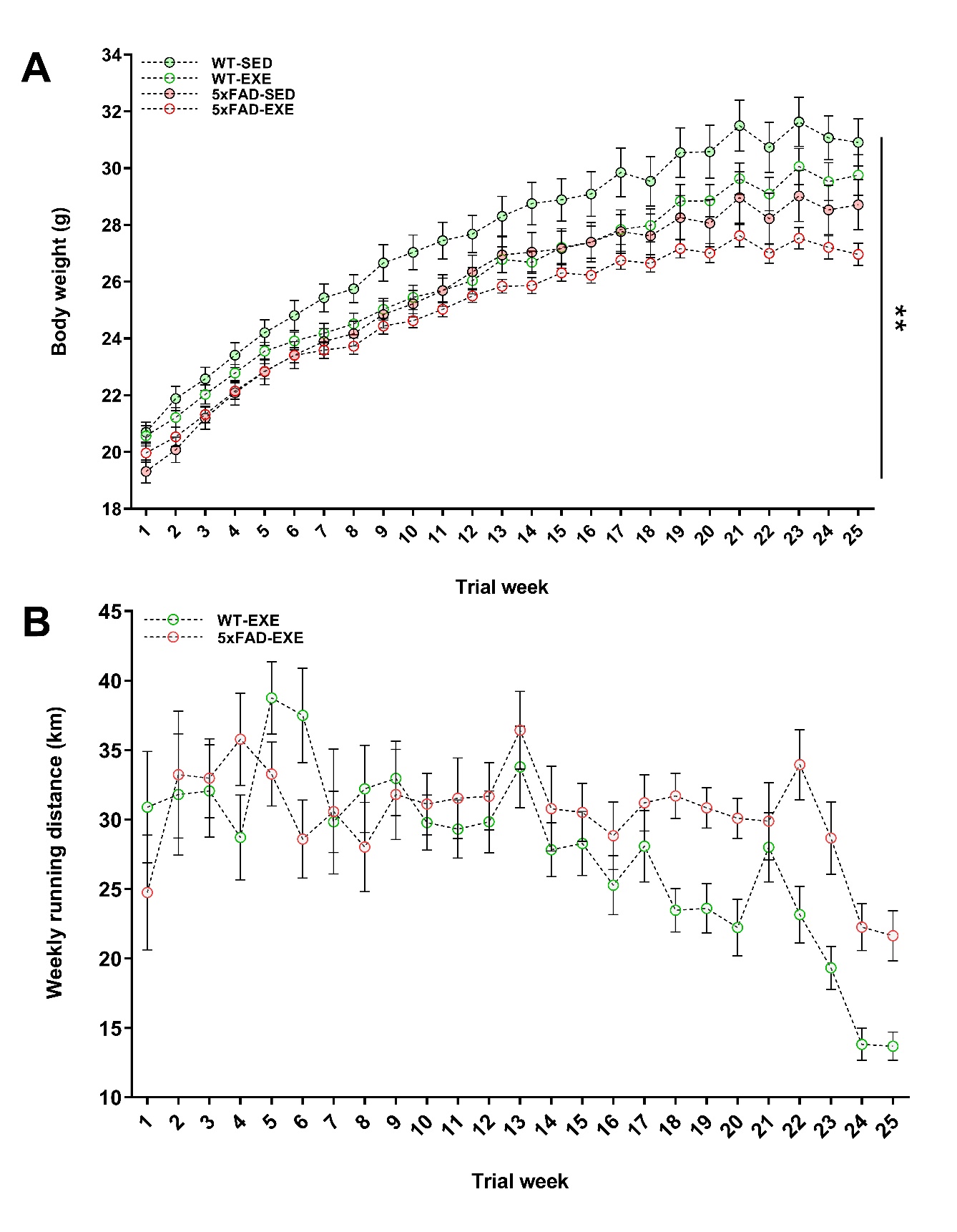
**Supplementary Figure 1.** Body mass and running activity evolution of WT and 5xFAD mice as measured on a weekly basis. **A** 5xFAD mice have significantly lower body weight than WT mice, whereas exercise slightly reduces body weight in all mice (*p <0.01, #p = 0.054). **B** Mice had free access to a running wheel from 1.5 to 7 months of age. The running distance was monitored for each mouse by using running counters. No significant differences were observed in the running distance between WT and 5xFAD mice (p = 0.166). Data is presented as mean ± SEM, one-way and two-way ANOVA for repeated measures, n = 20-22/group. « * » genotype effect, **p<0.01.


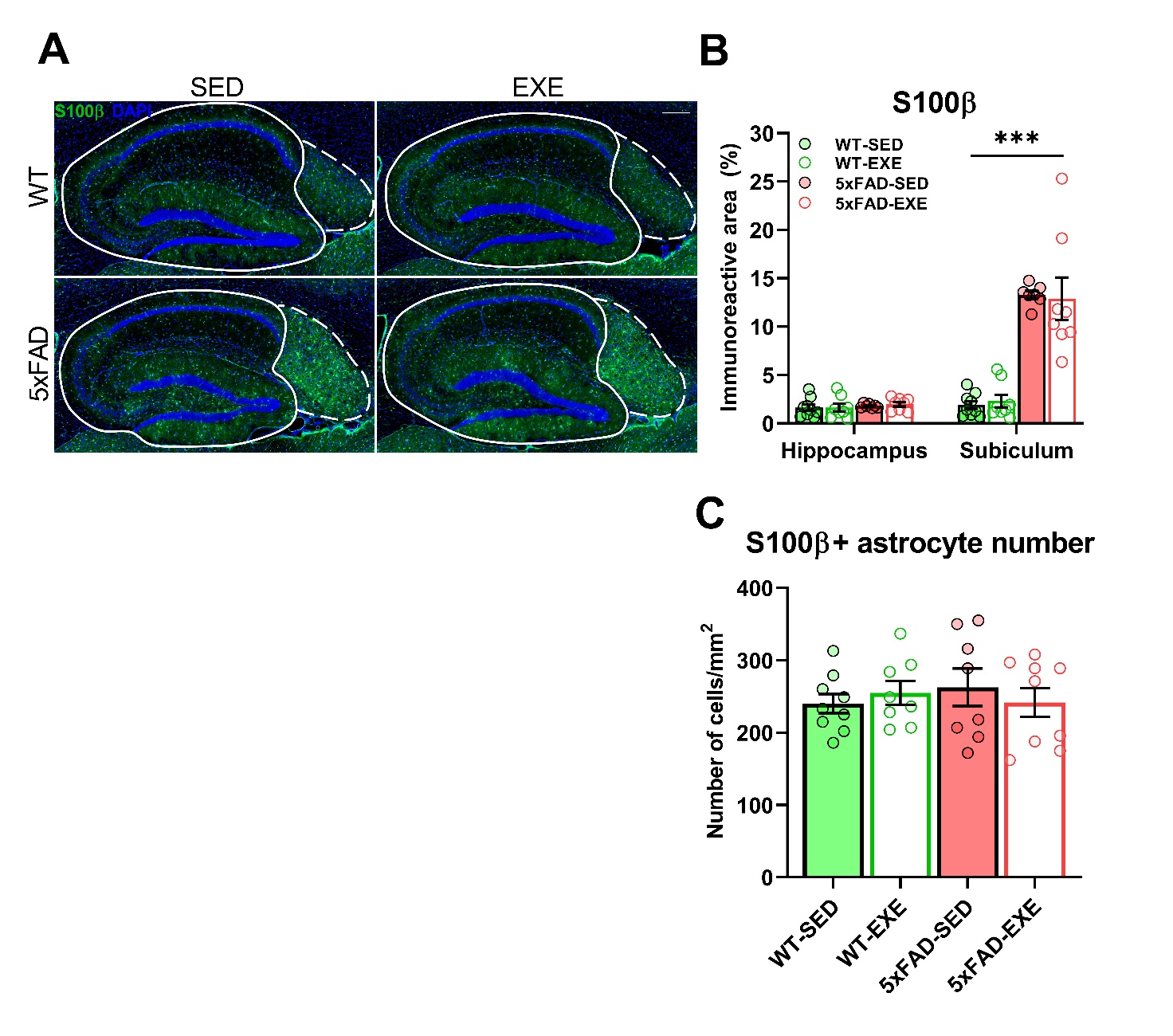


**Supplementary Figure 2.** Effect of voluntary physical exercise on S100β-positive astrocytes in hippocampi. Representative images of S100β-positive astrocytes (**A**, green) and DAPI (blue) in the hippocampal area of WT-SED, WT-EXE, 5xFAD-SED, 5xFAD-EXE mice. Solid and dotted lines represent quantified hippocampi and subiculum areas, respectively. Scale bars: 200 μm. S100β (B) levels were quantified by measuring the percentage of positive immunoreactive area in hippocampi and subiculum. Number of S100β -positive astrocytes per squared mm in hippocampi (**C**). All data are presented as mean (±SEM). 2-way ANOVA used to measure genotype and exercise effect between WT and 5xFAD mice. n=8/group, « * » genotype effect: ***p<0.001.

| *Antibody* | *Catalog number* | *Company* |
| --- | --- | --- |
| Amyloid β,clone WO-2, mouse | MABN10 | Millipore, Burlington, MA, USA |
| Iba-1, rabbit | 019-19741 | Wako, Tokyo, Japan |
| GFAP, rabbit | Z033429-2 | Dako, Glostrup, Denmark |
| GFAP, chicken | ab4674 | Abcam, Cambridge, MA, USA |
| S100β, rabbit | ab52642 | Abcam, Cambridge, MA, USA |
| GS, rabbit | ab73593 | Abcam, Cambridge, MA, USA |
| ALDH1L1, rabbit | ab87117 | Abcam, Cambridge, MA, USA |
| NeuN, rabbit | 711054 | Invitrogen, Waltham, MA, USA |
| Doublecortin, rabbit | 4604 | Cell Signaling Technology, Danvers, MA, USA |
| BDNF, rabbit | SAB2108004 | Sigma-Aldrich, St. Louis, MO, USA |
| BDNF, rabbit | ab108319 | Abcam, Cambridge, MA, USA |
| Synaptophysin, rabbit | MA5-14532 | Invitrogen, Waltham, MA, USA |
| PSD95, rabbit | 3450 | Cell Signaling Technology, Danvers, MA, USA |
| β-actin, mouse | A5441 | Sigma-Aldrich, St. Louis, MO, USA |
| Goat anti-rabbit IgG, Alexa Fluor 488 | A11008 | ThermoFisher Scientific, Waltham, MA, USA |
| Goat anti-mouse IgG, Alexa Fluor 568 | A11004 | ThermoFisher Scientific, Waltham, MA, USA |
| Goat anti-chicken IgG, Alexa Fluor 568 | A11041 | ThermoFisher Scientific, Waltham, MA, USA |
| Goat anti-rabbit IgG-HRP conjugate | 170-65-15 | BioRad, Hercules, CA, USA |
| Donkey anti-mouse IgG-Cy5 cojugate | 715-175-151 | Jackson Immuno Res. Lab., West Grove, PA, USA |
|  |  |  |
| *TaqMan primer (Amplicon Length)* | *Catalog number, ThermoFisher Scientific, Waltham, MA, USA* | |
| Gapdh (93) | Hs02758991_g1 |  |
| Gfap (75) | Mm01253033_m1 |  |
| S100β (69) | Mm00485897_m1 |  |
| Slc1a3 (71) | Mm00600697_m1 |  |
| Atp1b2 (80) | Mm00442612_m1 |  |
| Glul (112) | Mm00725701_s1 |  |
| Adh1l1 (73) | Mm03048957_m1 |  |
| Serping1_A1(103) | Mm00437835_m1 |  |
| Fkbp5_A1(74) | Mm00487406_m1 |  |
| Srgn_A1(111) | Mm01169070_m1 |  |
| S100f10_A2(71) | Mm00501457_m1 |  |
| Aif1(82) | Mm00479862_g1 |  |
| Bdnf (108) | Mm01334042_m1 |  |

**Supplementary Table 1.** List of antibodies and TaqMan primers used in this study.

|  | Cytokine concentration (pg/ml) | | | | Statistics | | |
| --- | --- | --- | --- | --- | --- | --- | --- |
|  | WT-SED | WT-EXE | 5xFAD-SED | 5xFAD-EXE | Genotype | Exercise | Interaction |
|  | (n=7) | (n=5) | (n=7) | (n=6) |  |  |  |
| IL-6 | 2.1 ± 0.2 | 2.5 ± 0.3 | 2.2 ± 0.2 | 2.6 ± 0.1 | 0.65 | 0.07 | 0.97 |
| IL-10 | 0.0 ± 0.0 | 0.8 ± 0.6 | 0.3 ± 0.2 | 0.9 ± 0.3 | 0.50 | 0.03^#^ | 0.61 |
| MCP-1 | 30.4 ± 1.6 | 32.3 ± 1.9 | 36.1 ± 2.0 | 38.5 ± 1.4 | 0.003** | 0.24 | 0.89 |
| IFNγ | 0.07 ± 0.04 | 0.16 ± 0.05 | 0.17 ± 0.05 | 0.20 ± 0.07 | 0.24 | 0.35 | 0.62 |
| TNFα | 0.6 ± 0.2 | 0.9 ± 0.2 | 1.0 ± 0.1 | 0.9 ± 0.2 | 0.46 | 0.66 | 0.36 |
| IL12p70 | 2.4 ± 0.3 | 3.0 ± 0.4 | 3.7 ± 0.4* | 2.3 ± 0.3^#^ | 0.37 | 0.33 | 0.02^§^ |

**Supplementary Table 2.** Effect of AD and voluntary physical exercise on cytokine levels in the hippocampus. Data are presented as mean (±SEM). 2-way ANOVA was used to measure genotype and exercise effect between WT and 5xFAD mice followed by unpaired t-test comparison in case of significant interaction between two factors (genotype*exercise). « * » genotype effect, « # » exercise effect, « § » interaction: *p<0.05, **p<0.01, ^#^p<0.05, ^§^p<0.05.

|  | ΔCт | | | | Statistics | | |
| --- | --- | --- | --- | --- | --- | --- | --- |
|  | WT-SED | WT-EXE | 5xFAD-SED | 5xFAD-EXE | Genotype | Exercise | Interaction |
|  | (n=5) | (n=5) | (n=5) | (n=5) |  |  |  |
| GFAP | 4.2 ± 0.1 | 4.1 ± 0.1 | 2.2 ± 0.1 | 2.4 ± 0.2 | <0.0001*** | 0.78 | 0.20 |
| S100β | 5.0 ± 0.1 | 4.9 ± 0.1 | 4.7 ± 0.1 | 4.7 ± 0.2 | 0.02* | 0.80 | 0.25 |
| Slc1a3 | 4.8 ± 0.2 | 4.8 ± 0.3 | 5.2 ± 0.2 | 5.2 ± 0.3 | 0.02* | 0.56 | 0.47 |
| Atp1b2 | 4.9 ± 0.1 | 5.2 ± 0.4 | 5.4 ± 0.1 | 5.4 ± 0.1 | 0.04* | 0.37 | 0.32 |
| Glul | 1.10 ± 0.08 | 1.06 ± 0.03 | 1.14 ± 0.07 | 1.27 ± 0.09 | 0.11 | 0.57 | 0.30 |
| ALDH1L1 | 7.13 ± 0.04 | 7.20 ±0.10 | 7.06 ±0.04 | 7.16 ± 0.16 | 0.43 | 0.27 | 0.66 |
| Serping1_A1 | 10.3 ± 0.3 | 10.8 ± 0.3 | 10.3 ± 0.3 | 10.8 ± 0.1 | 0.88 | 0.09 | 0.98 |
| Fkbp5_A1 | 7.1 ± 0.1 | 7.1 ± 0.3 | 6.7 ± 0.1 | 6.8 ± 0.1 | 0.02* | 0.91 | 0.88 |
| Srgn_A1 | 10.4 ± 0.4 | 11.0 ± 0.3 | 10.0 ± 0.2 | 10.2 ± 0.2 | 0.07 | 0.19 | 0.59 |
| S100A10_A2 | 7.52 ± 0.04 | 7.45 ± 0.02 | 7.19 ± 0.06** | 7.41 ± 0.06^#^ | 0.002** | 0.17 | 0.014^§^ |
| Aif1 | 9.2 ± 0.1 | 9.1 ± 0.1 | 7.7 ± 0.1 | 7.7 ± 0.1 | <0.0001*** | 0.96 | 0.63 |
| BDNF | 7.5 ± 0.1 | 7.5 ± 0.1 | 7.6 ± 0.1 | 7.5 ± 0.1 | 0.48 | 0.79 | 0.49 |

**Supplementary Table 3.** Effect of AD and voluntary physical exercise on mRNA expression of glial markers and BDNF in the hippocampus. Data are normalized by GAPDH C_T_ values and presented as mean (±SEM). 2-way ANOVA was used to measure genotype and exercise effect between WT and 5xFAD mice followed by unpaired t-test comparison in case of significant interaction between two factors (genotype*exercise). « * » genotype effect, « # » exercise effect, « § » interaction: *p<0.05, **p<0.01, ***p<0.001, #p<0.05, §p<0.05.

|  | Plaque associated | | Non-plaque associated | | Statistics | | |
| --- | --- | --- | --- | --- | --- | --- | --- |
|  | SED | EXE | SED | EXE | Plaque | Exercise | Interaction |
|  | (n=6) | (n=8) | (n=6) | (n=8) |  |  |  |
| N of Primary Branches | 6.8 ± 0.2 | 7.7 ± 0.3^#^ | 5.7 ± 0.3 | 5.7 ± 0.4 | <0.0001*** | 0.18 | 0.15 |
| Total Branch Length | 132 ± 19 | 111 ± 9 | 83 ± 6 | 78 ± 3 | 0.0003*** | 0.27 | 0.48 |
| Cell Radius | 27 ± 1 | 26 ± 1 | 23 ± 1 | 23 ± 1 | 0.0014** | 0.76 | 0.48 |
| Soma Area | 58 ± 7 | 88 ± 10^#^ | 39 ±3 | 38 ± 3 | <0.0001*** | 0.04^#^ | 0.03^§^ |
| Cell Area | 178 ± 27 | 181 ± 16 | 107 ± 8 | 103 ± 3 | <0.0001*** | 0.93 | 0.74 |

**Supplementary Table 4.** Morphological analysis of plaque-associated and non-plaque-associated GFAP-positive astrocytes in hippocampus of 5xFAD mice. Data are presented as mean (±SEM). 2-way ANOVA was used to measure plaque-association and exercise effect between sedentary (SED) and exercised (EXE) mice followed by unpaired t-test comparison in case of significant interaction between two factors (plaque*exercise). « * » plaque effect, « # » exercise effect, « § » interaction: **p<0.01, ***p<0.01, ^#^p<0.05, ^§^p<0.05.
